# A deconvolutional Bayesian mixing model approach for river basin sediment source apportionment

**DOI:** 10.1038/s41598-018-30905-9

**Published:** 2018-08-30

**Authors:** William H. Blake, Pascal Boeckx, Brian C. Stock, Hugh G. Smith, Samuel Bodé, Hari R. Upadhayay, Leticia Gaspar, Rupert Goddard, Amy T. Lennard, Ivan Lizaga, David A. Lobb, Philip N. Owens, Ellen L. Petticrew, Zou Zou A. Kuzyk, Bayu D. Gari, Linus Munishi, Kelvin Mtei, Amsalu Nebiyu, Lionel Mabit, Ana Navas, Brice X. Semmens

**Affiliations:** 1 School of Geography, Earth and Environmental Sciences, University of Plymouth, Plymouth, UK; 2 Isotope Bioscience Laboratory – ISOFYS, Ghent University, Gent, Belgium; 3 Scripps Institution of Oceanography, UC San Diego, La Jolla, CA USA; 4 Landcare Research, Palmerston North, New Zealand; 5 Soil and Water Department, Estación Experimental de Aula Dei (EEAD-CSIC), Zaragoza, Spain; 6 School of Environmental Sciences, University of Liverpool, Liverpool, UK; 7 Department of Soil Science, University of Manitoba, Winnipeg, Manitoba Canada; 8 Quesnel River Research Centre, University of Northern British Columbia, Prince George, British Columbia Canada; 9 Department of Geological Sciences, University of Manitoba, Winnipeg, Manitoba Canada; 10 College of Agriculture and Veterinary Medicine, Jimma University, Jimma, Ethiopia; 11 Nelson Mandela African Institute of Science and Technology, Arusha, Tanzania; 12 Soil and Water Management and Crop Nutrition Laboratory, Joint UN Food and Agricultural Organisation and International Atomic Energy Agency Division of Nuclear Techniques in Agriculture, Vienna, Austria; 13 Present Address: Catchment Systems, Sustainable Agriculture Sciences, Rothamsted Research, North Wyke, Okehampton UK

## Abstract

Increasing complexity in human-environment interactions at multiple watershed scales presents major challenges to sediment source apportionment data acquisition and analysis. Herein, we present a step-change in the application of Bayesian mixing models: Deconvolutional-MixSIAR (D-MIXSIAR) to underpin sustainable management of soil and sediment. This new mixing model approach allows users to directly account for the ‘structural hierarchy’ of a river basin in terms of sub-watershed distribution. It works by deconvoluting apportionment data derived for multiple nodes along the stream-river network where sources are stratified by sub-watershed. Source and mixture samples were collected from two watersheds that represented (i) a longitudinal mixed agricultural watershed in the south west of England which had a distinct upper and lower zone related to topography and (ii) a distributed mixed agricultural and forested watershed in the mid-hills of Nepal with two distinct sub-watersheds. In the former, geochemical fingerprints were based upon weathering profiles and anthropogenic soil amendments. In the latter compound-specific stable isotope markers based on soil vegetation cover were applied. Mixing model posterior distributions of proportional sediment source contributions differed when sources were pooled across the watersheds (pooled-MixSIAR) compared to those where source terms were stratified by sub-watershed and the outputs deconvoluted (D-MixSIAR). In the first example, the stratified source data and the deconvolutional approach provided greater distinction between pasture and cultivated topsoil source signatures resulting in a different posterior distribution to non-deconvolutional model (conventional approaches over-estimated the contribution of cultivated land to downstream sediment by 2 to 5 times). In the second example, the deconvolutional model elucidated a large input of sediment delivered from a small tributary resulting in differences in the reported contribution of a discrete mixed forest source. Overall D-MixSIAR model posterior distributions had lower (by ca 25–50%) uncertainty and quicker model run times. In both cases, the structured, deconvoluted output cohered more closely with field observations and local knowledge underpinning the need for closer attention to hierarchy in source and mixture terms in river basin source apportionment. Soil erosion and siltation challenge the energy-food-water-environment nexus. This new tool for source apportionment offers wider application across complex environmental systems affected by natural and human-induced change and the lessons learned are relevant to source apportionment applications in other disciplines.

## Introduction

### Context and aim

Fingerprinting and (un)mixing concepts are used widely across environmental disciplines for forensic evaluation of pollutant sources^1^. In freshwater and marine systems, this includes tracking the source of organic and inorganic pollutants in water^2–5^ and linking problematic sediment to soil erosion and specific land use sources^6–10^. It is, however, the complexity of ecological systems that has driven development of sophisticated Bayesian mixing models to appropriately represent inherent hierarchy and uncertainty in biogeochemical tracer data^11^.

In river basin sediment and contaminant mixing applications, the main parameters of interest are the proportions each ‘source’ contributes to a downstream ‘mixture’ within a river network wherein sources and mixtures are nested within the river basin and its sub- watershed structure. Knowledge of sediment source, transfer and residence time dynamics is critical to underpin sustainable land management for future food, water and energy security^12^ particularly for vulnerable communities threatened by socio-economic impacts of soil erosion^13,14^. Soil erosion threatens food security^15^ and associated siltation and pollution of river channels, lakes and reservoirs threatens river basin ecosystem service provision^16,17^, water security^18^, and hydro-electric power generation^19^. In response to challenges in tackling soil erosion and siltation problems worldwide, geochemical, radiochemical and isotopic fingerprinting techniques have developed considerably^20^. While these datasets capture real world sediment and pollutant source complexity in time and space, traditional statistical approaches used to select and treat these datasets can compromise source discrimination and apportionment. The power of geochemical, radiochemical and isotopic analytical techniques for sediment source and pollution apportionment may be more fully realized when these data are effectively coupled with new Bayesian modelling approaches.

In environmental and ecological mixing problems, a key advantage of Bayesian over conventional linear mixing models is their flexible likelihood-based structure which permits better representation of inherent variability in source and mixture tracer data due to environmental processes^21–23^. Bayesian models also enable existing knowledge, in the form of ‘prior’ probability distributions, to be combined with new tracer data to obtain updated ‘posterior’ probability distributions for parameters of interest. To date these models have primarily been used in ecology to evaluate diet composition, population structure, and animal movement^24–26^. In this context, a new Bayesian mixing model framework MixSIAR^11,27^, was developed. MixSIAR is not a single model but a general framework that can create many different models based on available data types and elective parameterisation^27^ with particular attention to the advantages of working with hierarchy in source and mixture data^11,27^.

While several river basin studies have successfully used versions of Bayesian mixing models to unmix sediment sources^21,22,28–34^, comparison of mixtures to sources is restricted to defined catchment units with potential loss of diagnostic detail with increasing scale. Here we present a step-change in application of Bayesian mixing models to river basin source apportionment problems with development of a hierarchical mixing model approach ‘*Deconvolutional MixSIAR*’ (D-MixSIAR). This extension of MixSIAR^11^ allows users to directly account for the ‘structural hierarchy’ of a river basin or watershed. Accordingly, this contribution aims to demonstrate the application of the D-MixSIAR approach to both geochemical and compound specific stable isotope (CSSI) tracer data in lowland agricultural (UK) and upland forested (Nepal) watershed settings. Herein we aim to outline the advantages of a new approach to stratifying source apportionment data by watershed that offers unique insight into complex river basin process dynamics along the soil-sediment continuum.

### Source apportionment in river networks: a new deconvolution approach

**Figure 1:**
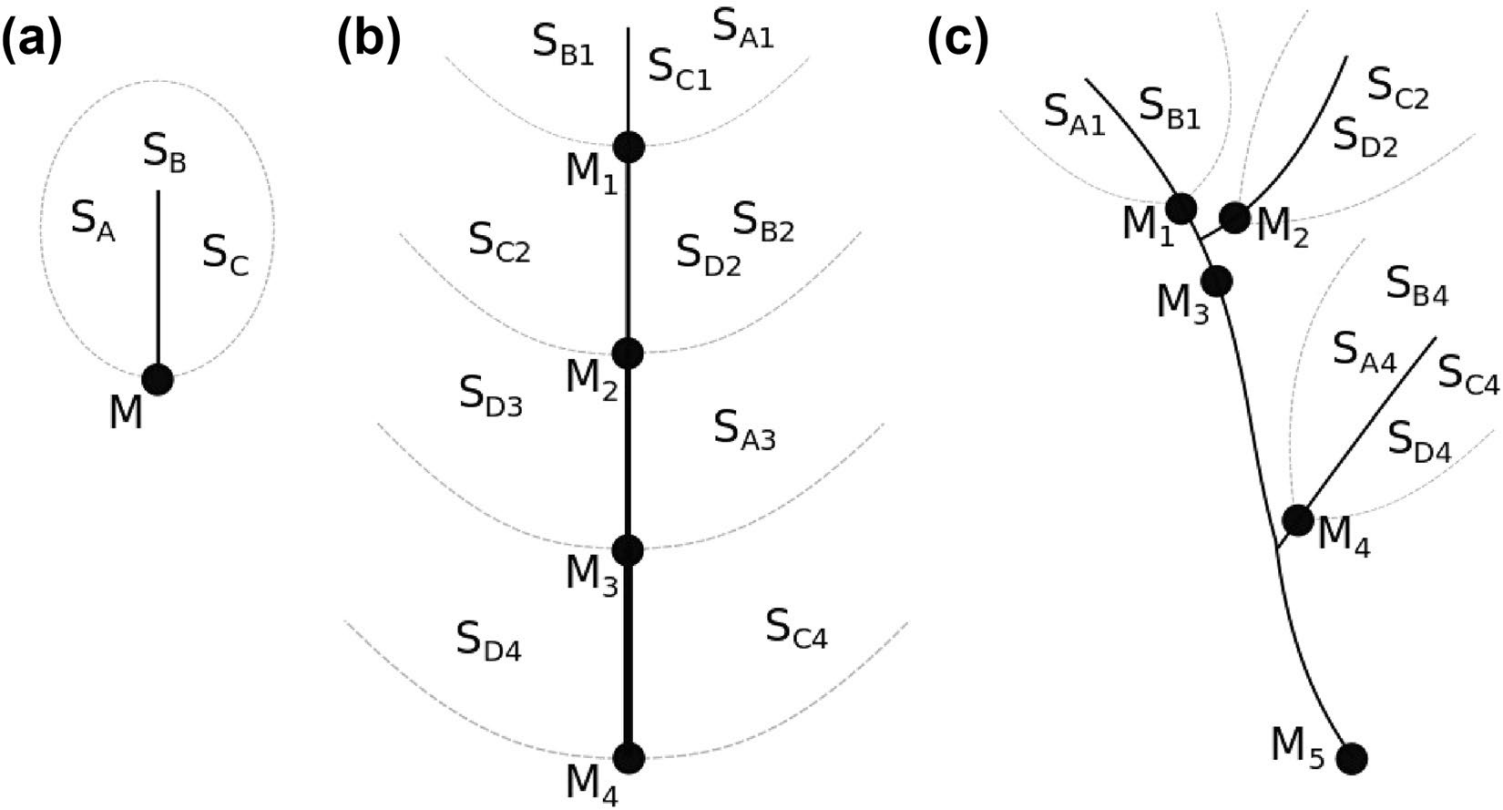
Example experimental designs demonstrating how MixSIAR apportions sources in hierarchical river networks. In all designs, rivers flow downward, filled circles represent nodes at which sediment mixture (Mix = M) samples could be collected, and dashed grey lines delineate watersheds (S) denoted by subscript numbers, and subscript letters indicate unique sources. (**A**) Simple watershed with three sources, S_A-C_, and one mixture location at the outflow, M. (**B**) Longitudinal system with four sources, S_A-D_, and multiple mixture locations at the outlet of each nested subwatershed, M_1–4_. (**C**) Distributed system with mixtures at the outflow of each of three subwatersheds, M_1_, M_2_, and M_4_, four sources (S_A-D_), as well as mixtures on the main channel: M_3_ and M_5_. (note: not all sources are present in all subwatersheds).

A key innovation of MixSIAR is the ability for the model to handle hierarchy in source and mixture data. In the river basin context, hierarchy is most obviously manifest in terms of river basin structure (e.g. nested or distributed watersheds from basin scale down in size to hillslope segments, Fig. 1). D-MixSIAR works by applying the MixSIAR mixing model sequentially to successive sediment mixture nodes (e.g. below a significant confluence) progressing downstream in a river network. Critically in D-MixSIAR, the source data for each node is stratified by sub-watershed. The full mathematical formulation details of MixSIAR are presented by Stock *et al*.^11^. Here we focus on the D-MixSIAR innovation and explain its application in the context of the two case studies. Full implementation code for D-MixSIAR is provided in Supplementary Information 1 (SI1).

A sediment mixture sampled from any node (with a target minimum three samples in total per node where feasible) in the river network is viewed as a mixture in relation to all upstream sources (Fig. 1a) and/or nodes (Fig. 1b and c), depending on location within the watershed structure. We propose two generic structural settings in which D-MixSIAR can be applied. Firstly, a longitudinal nested sub-watershed design (Fig. 1b) with, for example, four sources (S_A_, S_B_, S_C_, and S_D_) sampled from hillslopes across the river basin wherein sampling is stratified across nested sub-watershed 1 to 4, and sediment mixtures (at nodes M_1_, M_2_, M_3_ and M_4_) are collected at the outlet of each nested sub-watershed. The mixtures can theoretically be unmixed against the sources pooled across the entire watershed or sources nested within a specific sub-watershed by node (i.e. source data structured by sub-watershed). Upstream mixtures can also be included as ‘sources’ to downstream mixtures (i.e. M_3_ unmixes via M_2_, S_A3_, S_D3_ in Fig. 1b). Alternatively, the hypothetical sources might be stratified across sub-watersheds which are distributed throughout the wider river basin (Fig. 1c) wherein all mixture nodes contribute to river basin outlet sediment.

There are hence two ways that sediment mixtures might be unmixed against source materials. First, if the sub-watershed structure is ignored as in previous work, sediment mixtures can be unmixed using a watershed-wide source signature from samples pooled across sub-watersheds (e.g. for source D in Fig. 1b, combine S_D2_, S_D3_, and S_D4_ into S_D_, and so on for S_A_, S_B_ and S_C_, and then unmix M_4_ against S_A_, S_B_, S_C_, S_D_). We refer to this as the “*pooled MixSIAR*” approach. Alternatively, the *D-MixSIAR* method can be applied to conduct a sequential analysis using stratified upstream mixtures as sources to downstream mixtures. The individual sub-watersheds are analysed with conventional MixSIAR and the results integrated to deconvolute the upstream mixtures at each level in terms of primary source contributions determined from the level above. For example, in Fig. 1b, this is manifest through (1) unmixing M_1_ against S_A1_, S_B1_, S_C1_; (2) unmixing M_2_ against M_1_, S_B2_, S_C2_, S_D2_; (3) unmixing M_3_ against M_2_, S_A3_, S_D3_; and finally (4) M_4_ against M_3_, S_C4_, S_D4_. The D-MixSIAR routine (SI1) then recovers the overall contribution of the sources A-D to each of the mixtures by multiplying the estimated sub-watershed source proportions by the proportion each subwatershed contributes to the next mixture i.e. the proportion (p) of source B to mixture M_2_ is p_B1_*p_M1_ + p_B2_. Importantly, D-MixSIAR propagates uncertainty in the proportion estimates, because it estimates full posterior distributions for each of the proportions. This principle can be extended to cover the nested scenario (Fig. 1c).

## Methods

### Study watersheds

**Figure 2:**
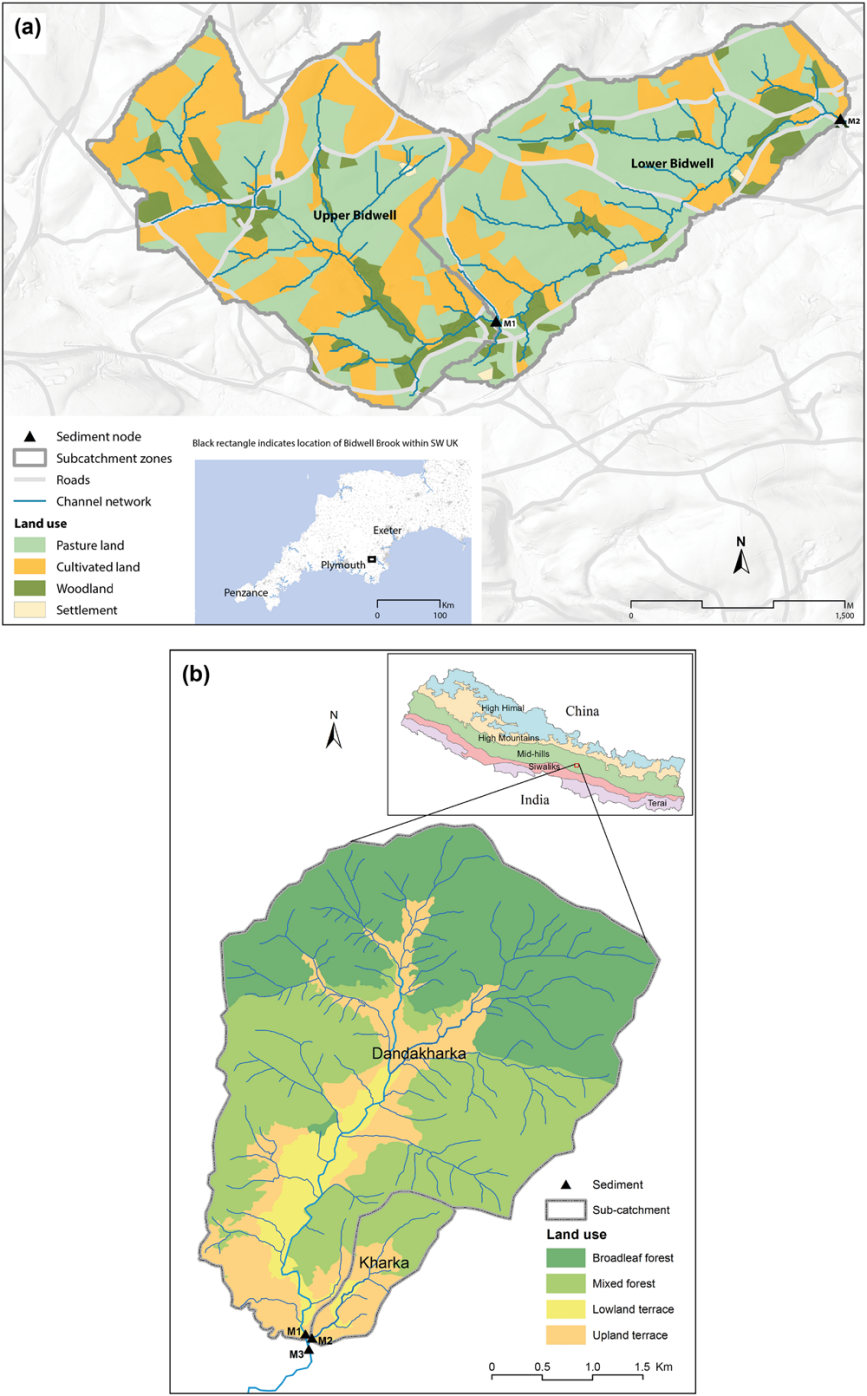
Study watersheds. (**a**) Bidwell Brook, south west UK and (**b**) Upper Chitlang, Nepal where M1–M3 refers to the sediment mixture sampling nodes (see Fig. 1) and land use cover relates to identified sources.

To evaluate D-MixSIAR in the context of longitudinal (Fig. 1b) and distributed (Fig. 1c) watershed systems, source apportionment data were collected from (1) a lowland mixed agricultural catchment in southwest UK (Bidwell Brook) and (2) an upland mixed forest and agricultural watershed in Nepal (Upper Chitlang) (Fig. 2a and b). The former utilised major and minor element geochemistry, the latter used CSSIs^35^.

Bidwell Brook watershed (12 km^2^) has a maritime temperate climate receiving ca 1000 mm rainfall per annum with a notable wet autumn and winter period. Derived from a sedimentary substrate, the soil is a freely draining loam with moderate risk of damage by farm machinery (surface compaction and subsurface plough pan). Land use across the watershed is a mixture of pasture (P) and cultivated (CU) land (Table 1) where cultivated land is in rotation between i.e. maize, wheat, barley, root crops and ley grass. Upper and lower zones of the watershed have two notable differences in land management practice. Cultivation in the upper section of the study area is undertaken under certified organic practice i.e. no mineral fertiliser application and fields are generally buffered by grass strips. Pasture fields in the upper catchment are predominantly permanent (PP) on steeper slopes and used for grazing of dairy cattle with the remainder in rotation (RP). In the lower section of the watershed, cultivated land is farmed conventionally and the greater proportion of pasture fields is in rotation (RP).

**Table 1:** Sampled source distributions in (sub) watersheds of the Bidwell watershed (UK) and the Chitlang watershed (Nepal) where P = pasture, CU = cultivated land, RM = road-derived material and CB = channel bank, BLF = broad leaf forest, MF = mixed forest, LL = lowland terraces and UP = upland terraces. Mix 1–2 (Bidwell) and Mix 1–3 (Chitlang) refer to Fig. 1b and c respectively.

Watershed unit	Model Component	Source land cover (%)				Area (km^2^)
		P	CU	RM	CB	
Upper Bidwell	Mix 1	42	47	<1%	<1%	4.20
Lower Bidwell	Mix 2	51	38	<1%	<1%	7.90
		**BLF**	**MF**	**LL**	**UP**	
Dandakharka	Mix 1	41	38	6	16	14.4
Kharka	Mix 2	n/a	48	7	44	1.00
Upper Chitlang	Mix 3	38	36	6	19	15.4

Note the remainder of land cover in Bidwell Brook is largely stable woodland which was not included in the model.

The Upper Chitlang watershed receives ca 1600 mm rainfall per year 77% of which falls during a monsoon period from June to September. Soil is classified as a Cambisol that has developed over sedimentary alluvium substrate. In this study, two sub-watersheds that flow to a common tributary were selected, the Dandakharka (14 km^2^) and Kharka (1 km^2^) (Fig. 2). At the time of sampling, the Dandakharka had four dominant land uses (Table 1) i.e. broadleaf forest (BLF), mixed forest (MF), lowland agricultural terraces (LL) and upland agricultural terraces (UP). Rice is the dominant crop on lowland terraces during monsoon season with flood irrigation. Wheat and commercial vegetables are grown in the winter season. Upland terraces are rain fed with maize as dominant crop, intercropped with finger millet and vegetables. Upland terraces are more scattered and fragmented. Land use in the Kharka was similar but without BLF. Forest types were based on dominant vegetation but it should be noted that MF zones were formerly natural BLF so remnant and young broadleaf trees are also present. Leaf litter collection is common practice among farmers for livestock bedding material.

### Source and mixture sampling and analysis

In both systems, sources were sampled in separate groups for each sub-watershed, i.e. sources were stratified. Depending on source extent, between 10 and 30 composite samples (each a combination of ca. 15 random samples from the site) were collected from the upper 20 mm of soil at sites that represented the source class. For the Bidwell site, total source sample numbers were 32, 34 and 38 for CU, PP and RM respectively. For the Chitlang site, total source samples were 11, 13, 14 and 15 for BLF, LL, MF and UP respectively. The Bidwell study included samples of channel bank material scrapes (n = 32) where exposed. Source materials were air dried (<45 °C) and disaggregated. For CSSI analysis, samples were sieved to <2 mm as the signatures are independent of grain size^35^. For geochemical analysis, samples were sieved to <63 µm to minimise grain size effects on tracer signals^36^.

At each node (M1 and M2) in the longitudinal model of the Bidwell watershed, channel-bed deposited sediment^37^ (n = 5 and 3 for each node respectively) and time-integrated suspended sediment samples^38^ (n = 1 and 3) were collected through the autumn/winter period (access to suspended sediment samplers was restricted by high flow at node 1). Since we hoped to gain inference into potential contrasting source dynamics of these two sediment budget components, we parameterised sediment mixture ‘type’ (bed versus suspended) as a factor in MixSIAR^11^ noting that while the time-integrated sampler numbers were limited, the nature of collection over a series of events underpinned representativeness. In the Chitlang distributed model, time-integrated suspended sediment samples^38^ were collected at each of the three nodes (n = 9, 8 and 8) where nodes M1 and M2 represented the sub-watersheds and node M3 represented the resulting mix in the higher order channel after the confluence. Time-integrated samples were collected across three periods in the wet season (i.e. early, mid and late), as we expected differences in sediment proportions due to seasonally-dependent land use practices. Since we hoped to gain inference on seasonal effects on the mixing process, we parameterised season as a factor. The “residual only” error formulation^11,23^ was used due to limited mixture sample numbers at the nodes with application of factors, which is likely to be a common scenario in river basin applications. All sediment samples were dewatered through a combination of settling and centrifugation prior to freeze drying and disaggregation and sieving as above.

All samples from the Bidwell watershed were analysed for major and minor element geochemistry by Wave Length Dispersive X-Ray Fluorescence (WD-XRF; PANalytical Axios Max; OMNIAN application) as pressed pellets. All samples from the Chitlang watershed were prepared for CSSI analysis (Fatty Acids, FAs) by capillary gas chromatography-combustion-isotope ratio mass spectrometry (GC-C-IRMS; Trace GC Ultra interfaced via a GC/C III to DeltaPLUS XP, Thermo Scientific, Bremen, Germany) as described by Upadhayay *et al*.^39^.

### Tracer selection

In light of Bayesian modelling advantages, we adopted a simplified tracer screening, i.e. range test, process. This step away from relying on ‘objective’ statistical techniques for selecting tracers was (1) based on recent proposals that a biogeochemical basis for selection is logically more appropriate^40,41^ and (2) because the covariance structure of MixSIAR^11^ handles redundancy so tracer selection by discriminant function analysis is not required, and might reduce discrimination or lead to erroneous outcomes. MixSIAR accounts for uncertainty in source and mixture data due to sampling or natural variability in the field. Two mechanisms accomplish this: fitting source tracer values within the model^11,27^, and specifying a distribution for the mixture data (i.e. an error structure^23^). In the Bayesian framework, striving to eliminate ‘redundant’ tracers is secondary to ensuring that tracers used are behaving independently and conservatively in the environment and indeed inclusion of even weak tracers can only improve model representation (cf.^42^). This differs from recent observations made using linear unmixing models^43^ because of the way the model is formulated.

For all tracers, boxplots were produced for each set of sources and associated mixtures and mixture data assessed to see if they largely fell within or outside of the sources. Tracers that were clear poor performers, i.e. the mix values were largely outside the source range, were removed. Borderline tracers were retained based on the principle that Bayesian model convergence statistics^25^ are the best assessment of fit. The approach implicitly assumed that sources and mixtures were representative and comparable and that correction factors for the tracers used were unnecessary^36,44^. MixSIAR assumes that mixture tracer data are normally distributed, which is appropriate because they are weighted combinations of the source means. Therefore, the central limit theorem applies, and a mixture of sources should be approximately normally distributed even if the sources are not^11^. Hence for the geochemical datasets, Exploratory Data Analysis (EDA) tools (histograms) were used instead of strict tests of normality. In addition, geochemical data that passed the range test were scrutinised for potential non-conservativeness in terms of fluvial sorting and biogeochemical process based on published data regarding environmental behaviour. For the CSSI datasets, basic descriptive statistics i.e. mean, and standard deviation (SD) were used to characterize the variation of δ^13^C-FA values within land uses and to describe data distributions. One-way analysis of variance (ANOVA) was used to assess the level of significance (p < 0.05) of δ^13^C-FA (C_22_–C_32_) in the Chitlang watershed. Before ANOVA, all variables were checked for a normal distribution and homogeneity of variance. In case of significant ANOVA, means were compared by Tukey’s honestly significant differences (HSD) (p < 0.05).

### Mixing model implementation

MixSIAR is implemented as an open-source R package^27^. Full details of the mathematical formulation of MixSIAR are provided by Stock *et al*.^11^. Model performance has previously been validated^23^ with simulation tests and 16 literature datasets, and the R package includes 11 examples with data that replicate published analyses^27^.

For the Bidwell study, MixSIAR was firstly formulated for each node separately with 15 tracers which passed the range test (Na, Mg, Al, P, S, Cl, Ca, Cr, Co, Cu, Ni, Ga, Rb, Nb, Ce) using a residual error term and sediment type as a factor and an uninformative prior (i.e. Dirichlet hyperparameters all set to 1 – see Stock *et al*.^11^). In a second model run we specified an informative prior wherein the Dirichlet hyperparameter for channel bank sources set at 0.01 because of potential overlap between channel bank and topsoil source signatures due to shared mixed weathering profiled origin^44^ and limited field evidence of active bank retreat. The influence of a Dirichlet prior increases with fewer data points, greater source data variance, and poorer separation between source signatures^11^. For the Chitlang study, concentration-dependent^45^ MixSIAR for each node was formulated with the δ^13^C values of six even FAs (C_22_–C_32_) for node M1 and three FAs (C_24_, C_26_ and C_28_) for node M2 using a residual error term, season as a factor and using an uninformative prior. The node posterior proportion contributions in both examples were then deconvoluted using the D-MixSIAR framework (section 2.1, SI1). For comparison to non-deconvolutional approaches, MixSIAR was subsequently formulated in both study watersheds using all source material signatures for each class pooled across both watershed units and all node mixtures from the systems compared to their respective pooled sources. Here the model was run with an uninformative prior (i.e. Dirichlet hyperparameters all set to 1).

In all MixSIAR model runs, the Markov Chain Monte Carlo (MCMC) parameters were generally set as follows: chain length = 1000000, burn = 700000, thin = 300, chains = 3. Convergence of all models was evaluated using the Gelman-Rubin diagnostic, rejecting model output if >5% of total variables was above 1.05, in which case chain length was increased.

## Results and Discussion

### Source tracer properties

**Figure 3:**
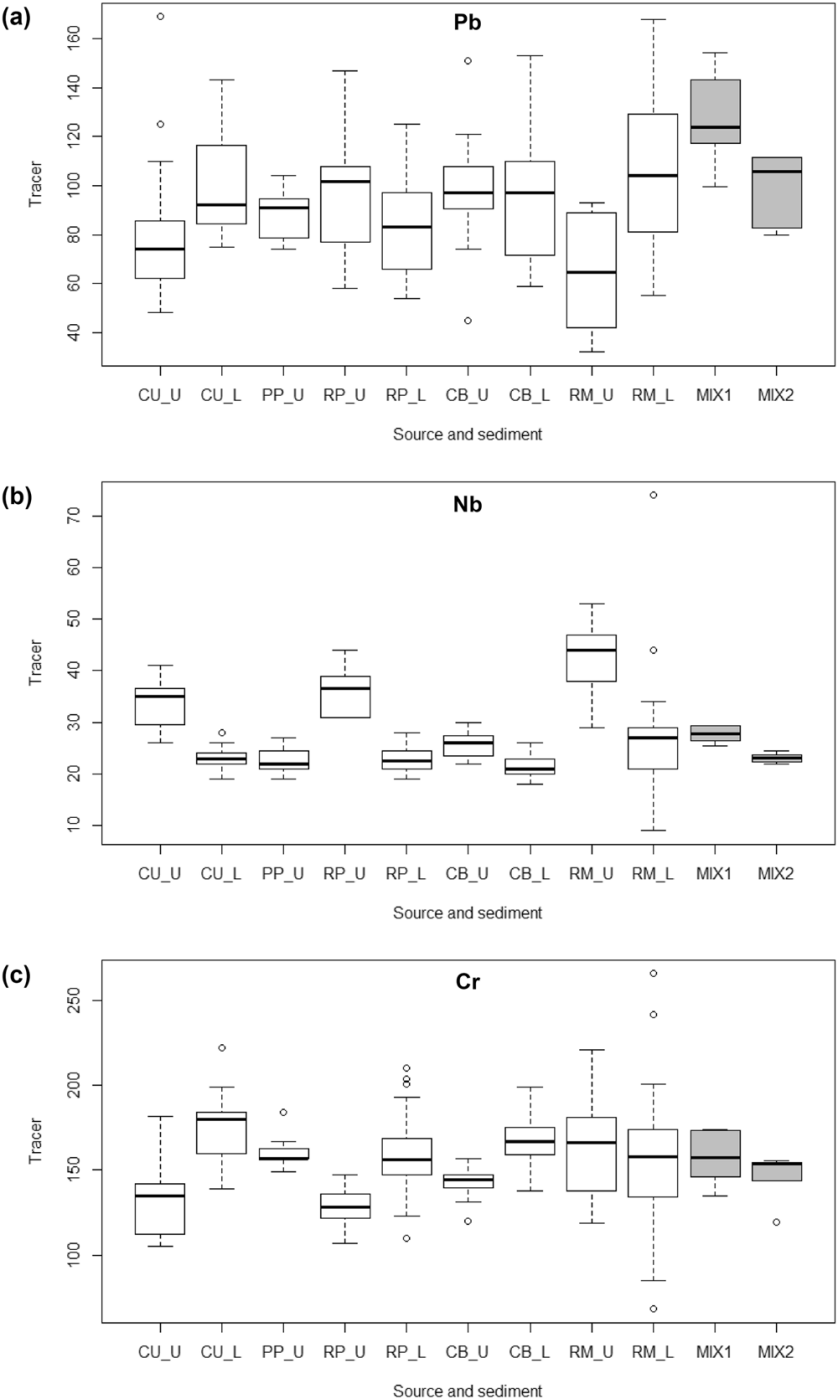
Example tracer distributions for (**a**) non-conservative (Pb), (**b**) weathering-controlled (Na) and (**c**) land management-amended (Cr) tracer properties in Bidwell Brook, in UK where Mix.1 and Mix.2 relate to sediment sampled at nodes M1 and M2 (See Fig. 1 and text for details). In box plots, median is shown by central line, interquartile range by box, range by whiskers with circles indicating outliers.

For the Bidwell case, of 26 major and minor geochemical elements measured in source and mixture samples of each node for D-MixSIAR, boxplot-based range testing (full data in Supplementary Information 1 (SI 1), examples in Fig. 3) led to elimination of seven elements (Pb, Ba, Br, Zn, Mn, Fe, Sr and Zr) based on lack of coherence between mix and source in either one or both nodes. Exclusion warrants some geochemical explanation^46^. Non conservative behaviour of Br can be explained by its propensity to form highly soluble salts^47^. Trace metals Pb and Zn concentrations were augmented in mixture sediment (Fig. 3a) suggesting an influence of sediment water interaction, noting high concentrations of these elements in road-related material) with a potential grain size control^48^. Barium, and Sr by association^47^, was also augmented in sediment either as a consequence of co-precipitation of weathering product with Fe and Mn^47^ or mineralogical controls on composition due to preferential sorting. Iron and Mn also failed the range test implying a degree of environmental mobility and co-precipitation on fine particles^48^. Elimination of these range tested elements also raised concern regarding wider sorting, i.e. textural, controls on mineral composition i.e. changing proportions of silt *versus* clay minerals in mixtures which has been shown to exert a strong influence on sediment Fe, K, Si, Ti and Zr concentrations^49^. Given overlap with range test failures in Fe and Zr, these elements were also withdrawn on this basis. The viability of the remaining elements was underpinned by their known variability in the soil profiles due to weathering and mixing of soil horizons by cultivation (e.g. Na, Ca, Mg, Rb, Nb) (Fig. 3b) or association with anthropogenic amendments to soil^50^ (e.g. Co, Cr, Cu) (Fig. 3c). For pooled MixSIAR, a wider range of mixture properties resulted in fewer exclusions: Ba, Br, Pb and Zn plus Fe, K, Ti and Si. Some elements retained might be considered less conservative in other systems (e.g. P, S and Cl). Here they were included due to clarity in discrimination between sources (SI 1) and limited scope for transformation at the scale of study^51^. Use of different elemental combinations had a notable outcome on posterior distributions and challenged convergence statistics, as discussed below.

**Figure 4:**
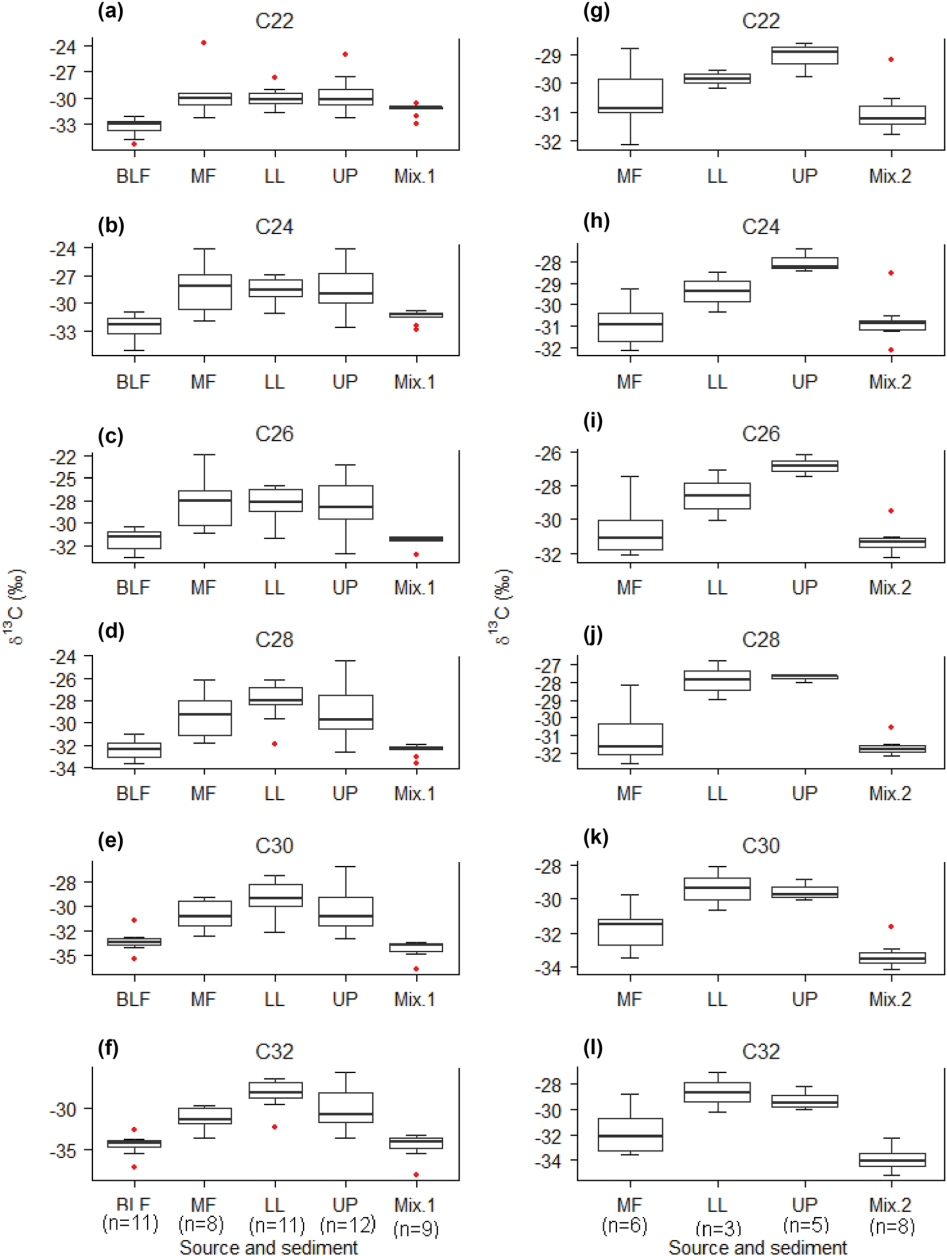
Distribution of δ^13^C (‰) values of FAs (C_22_–C_32_) in sources and sediments within Dandakharka (**a**–**f**) and Kharka (**g**–**l**) subcatchments (for M1 (Mix1) and M2 (Mix2) see detail in Fig. 2b). In box plots, median is shown by central line, interquartile range by box, range by whiskers with circles indicating outliers. Figures in the parenthesis in x-axis indicates the number of samples. Sources: broadleaf forest (BLF), mixed forest (MF), lowland terraces (LL) and upland terraces (UP).

Elimination of short-chain as well as unsaturated fatty acid (<20C atoms) tracers from the CSSI dataset was based on their biogeochemistry and behaviour in the soil and sediment environment^39^. These FAs are biosynthesised by both plants and microorganisms and are not stable in soil and sediment. In contrast, saturated long-chain FAs (>20C atoms) are exclusively biosynthesised by higher plants and are more stable than short-chained FAs^52^. Consequently, δ^13^C values of saturated long-chain FAs were hypothesised to characterise land uses defined by vegetation cover and it was assumed, based on the above, that they behaved conservatively in the watershed environment. Moreover, the range test was used to assess possible corruption in the isotopic value of FAs in the sediment (Fig. 4). In contrast to node M1, the δ^13^C values for node M2 mixtures fell outside the source range for chain length C_22_, C_30_ and C_32_. This potentially implies poor characterisation of spatial variability of sources, especially in mixed forest due to inaccessibility or non-conservative behaviour. A variety of alternative factors are likely responsible for the variation of stable isotope signature of long-chain FAs including differences in the legacy of inputs from previous vegetation^53^ and/or in current input from understory species^54^.

### Source apportionment: Deconvolutional MixSIAR versus Pooled MixSIAR

#### Longitudinal, lowland agricultural watershed

D-MixSIAR and pooled MixSIAR posterior distributions, with the uninformative prior, showed differences in source apportionment for the Bidwell watershed (Table 2). In general, pooled MixSIAR apportionment data showed largely similar proportions across all of the identified factors i.e. node M1, node M2, bed sediment and suspended sediment with the exception of substantially lower channel bank contribution at node 1. Cultivated land was designated the dominant source in node 1 (51%) and node 2 (44%). In contrast, D-MixSIAR reported ca 10% cultivated soil input to node M1 and a greater proportion of pasture soil input (from both permanent and rotational pasture). Cultivated soil in the upper catchment is farmed under organic certification with extensive buffering that limits structural connectivity^55^ to the stream. In contrast, steeper permanent pasture fields with notable poaching by livestock were observed to be strongly connected to the road network which drains directly into the brook at node M1. Additional structure in source data (i.e. separation of rotational and permanent pasture) led to tighter source terms and a different posterior outcome. Given the influence of farming practice on the cultivated soil signature in the upper watershed compared to the lower and the additional pasture categories when stratified by sub-watershed, we argue that D-MixSIAR gives a more credible posterior distribution in this case. Both pooled MixSIAR and D-MixSIAR posterior distributions imply a greater contribution of channel bank material to the bed sediment component in node M2 compared to suspended sediment which can be linked to localised channel bank slumping.

**Table 2:** D-MixSIAR and pooled MixSIAR source apportionment data for the Bidwell watershed for (a) model runs with uninformative prior where CB is channel bank, CU is cultivated soil, PP is permanent pasture (node M1 only), RM is rotational pasture (i.e. cultivated at some point in the past), P is the former 2 combined (for pooled MixSIAR), and RM is material sampled from the roads.

Model unit	Source	D-MixSIAR (factor = type)		Pooled-MixSIAR (factors = node and type)
		*Susp*. *Sed*	*Bed sed*.	
Upper Bidwell (Node M1)	CB	0.19 ± 0.13	0.14 ± 0.10	0.05 ± 0.06
	CU	0.11 ± 0.07	0.10 ± 0.07	0.51 ± 0.16
	PP	0.39 ± 0.13	0.47 ± 0.10	0.26 ± 0.15 (P)
	RP	0.15 ± 0.10	0.11 ± 0.07	
	RM	0.16 ± 0.05	0.17 ± 0.04	0.18 ± 0.11
Lower Bidwell (Node M2)	CB	0.22 ± 0.11	0.32 ± 0.09	0.36 ± 0.15
	CU	0.21 ± 0.10	0.19 ± 0.09	0.44 ± 0.15
	PP	0.27 ± 0.10	0.27 ± 0.07	0.09 ± 0.10 (P)
	RP	0.12 ± 0.04	0.13 ± 0.07	
	RM	0.18 ± 0.10	0.10 ± 0.03	0.11 ± 0.08
Bed sediment (both nodes M1 and M2)	CB	n/a	n/a	0.24 ± 0.17
	CU	n/a	n/a	0.39 ± 0.19
	P	n/a	n/a	0.16 ± 0.13
	RM	n/a	n/a	0.21 ± 0.16
Suspended sediment (both nodes M1 and M2)	CB	n/a	n/a	0.15 ± 0.13
	CU	n/a	n/a	0.40 ± 0.20
	P	n/a	n/a	0.25 ± 0.18
	RM	n/a	n/a	0.20 ± 0.15

Note pooled MixSIAR delivers posterior distributions according to either node or type as factors.

Previous studies have debated mixing model results regarding channel bank contributions^56^ and the potential influence of overlapping source signatures^44^. The use of an informative prior based on observational evidence offers an additional analysis tool for users wherein the posterior output will represent an optimal compromise between the prior distribution and the data. When the arbitrary informative prior implying limited channel bank erosion was used, the proportion of channel bank material in model outputs (Table 3) was reduced by 10–20%. For node M1, bank contribution was reduced to zero in the D-MixSIAR posterior alongside a 10% increase in the topsoil sources. For node M2 channel bank contribution to suspended sediment was reduced to 4% in exchange for topsoil sources but for bed material the channel bank contribution was still notable at 18% (Table 3). Informative priors are more likely to be based on circumstantial evidence in river basin science as opposed to direct evidence as used in ecology (e.g. stomach contents informing animal diet analysis). In addition to observation-based inference, as in the above example, informative priors might readily be generated through (1) land use cover data overlain by erosion risk assessment^57^, (2) qualitative watershed walkover data^58^, (3) stream sediment load data, and (4) results from prior tracer studies^59^. Whatever users adopt to explore sensitivity of model output to prior information, it is critical that model outputs with uninformative and informative priors are compared and contrasted. In this case, while user inference was that channel bank erosion was minimal, model outputs demonstrated that this source was in fact an important contributor in the lowland reaches, especially to bed-stored material.

**Table 3:** D-MixSIAR and pooled MixSIAR source apportionment data for the Bidwell watershed for model runs with an informative prior regarding limited channel bank input.

Model unit	Source	D-MixSIAR (factor = type)		Pooled-MixSIAR (factors = node and type)
		*Susp*. *Sed*	*Bed sed*.	
Upper Bidwell (Node M1)	CB	0.00 ± 0.03	0.00 ± 0.02	0.02 ± 0.03
	CU	0.13 ± 0.08	0.13 ± 0.07	0.55 ± 0.19
	PP	0.51 ± 0.10	0.55 ± 0.08	0.24 ± 0.19 (P)
	RP	0.19 ± 0.11	0.15 ± 0.08	
	RM	0.16 ± 0.05	0.17 ± 0.04	0.19 ± 0.13
Lower Bidwell (Node M2)	CB	0.04 ± 0.06	0.18 ± 0.11	0.30 ± 0.15
	CU	0.24 ± 0.11	0.23 ± 0.14	0.51 ± 0.18
	PP	0.37 ± 0.13	0.34 ± 0.07	0.07 ± 0.12 (P)
	RP	0.21 ± 0.10	0.15 ± 0.08	
	RM	0.13 ± 0.04	0.11 ± 0.03	0.12 ± 0.09

#### Distributed upland forested watershed

In the Nepalese forested catchment example, posterior distributions from MixSIAR run node-by-node (i.e. the raw ingredients of the D-MixSIAR prior to deconvolution) for node M1 (Table 3) implied that broadleaf forest contributed the majority (50–70%) of sediment to the Dandakharka stream network in all seasons, with a lesser but notable contribution from mixed forest (~20–35%) and the remainder from upland cultivation. In the Kharka subwatershed, posterior distributions implied that mixed forest dominated inputs to node M2 (Table 3). Raking leaf litter in the community managed forest is common practice^60^ and is used as bedding material for livestock and we surmise that this disturbance of the forest floor litter and O-horizon leads to enhanced overland flow on the steep forest slopes. When node M3 was unmixed against nodes M1 and M2, the smaller system (Kharka) was shown to have contributed a disproportionate amount of the load (Table 3) for sub-watershed area i.e. ~25% of load from 6.5% of the total watershed. The Dandakharka watershed is less disturbed and larger than the Kharka.

Application of pooled MixSIAR to node M3 (Table 4) implied that broadleaf forest contributed the majority (~70–80%) of sediment to node M3 in all seasons, with minor contribution from mixed forest (~10–15%, Table 4). D-MixSIAR, however, based on deconvolution of the above individual model runs (Table 4) revealed clear differences in watershed-specific controls on sediment source dynamics because it took into account the contribution of each sub-watershed’s stratified sources. Deconvolution of the posterior distributions gave substantially different results than the pooled MixSIAR approach. Broadleaf forest contributions to node M3 were markedly reduced in favour of mixed forest contributions (Table 3). Despite both broadleaf and mixed forest areas being located on similar slope units, the mixed forest source was observed to have greater levels of disturbance due to proximity to settlements and its location in the drier, lower and more connected part of watershed (patches of forest fires were also observed in MF during field visit).

**Table 4:** Temporal variability in relative contributions (mean ± SD) of sediment sources within individual sub-catchment and sub-catchments contribution to sediments downstream to confluence using MixSIAR node-by-node i.e. the raw ingredients of the D-MixSIAR prior to deconvolution.

Catchment	Source	Season		
		EW (April-June)	MW (July-Aug)	LW (Sep-Oct)
Dandakharka [Node M1]	BLF	0.70 ± 0.11	0.58 ± 0.12	0.50 ± 0.12
	MF	0.19 ± 0.1	0.28 ± 0.13	0.35 ± 0.14
	LL	0.03 ± 0.03	0.03 ± 0.04	0.03 ± 0.04
	UP	0.08 ± 0.08	0.11 ± 0.13	0.12 ± 0.14
Kharka [Node M2]	MF	0.75 ± 0.17	0.87 ± 0.14	0.76 ± 0.20
	LL	0.12 ± 0.12	0.06 ± 0.09	0.14 ± 0.17
	UP	0.13 ± 0.11	0.07 ± 0.10	0.09 ± 0.11
Confluence [Node M3]	Dandakharka	0.74 ± 0.17	0.83 ± 0.17	0.78 ± 0.2
	Kharka	0.26 ± 0.17	0.17 ± 0.17	0.22 ± 0.2
**Model type**	**Sources**	**Season**		
		**EW (April-June-)**	**LW (July-Aug)**	**MW (Sept-Oct)**
Deconvolutional MixSIAR [Node M3]	BLF	0.52 ± 0.14	0.41 ± 0.13	0.46 ± 0.15
	LL	0.05 ± 0.05	0.05 ± 0.06	0.04 ± 0.05
	MF	0.33 ± 0.13	0.42 ± 0.15	0.42 ± 0.17
	UP	0.09 ± 0.07	0.12 ± 0.13	0.09 ± 0.11
Pooled MixSIAR [Node M3]	BLF	0.79 ± 0.07	0.76 ± 0.08	0.81 ± 0.08
	LL	0.03 ± 0.03	0.03 ± 0.04	0.03 ± 0.03
	MF	0.12 ± 0.08	0.14 ± 0.10	0.11 ± 0.08
	UP	0.06 ± 0.05	0.07 ± 0.07	0.06 ± 0.06

(Seasons: EW = early wet, LW = late wet and MW = mid-wet and sources: BLF = broad leaf forest, MF = mixed forest, LL = lowland, and UP = upland) (b) pooled MixSIAR versus D-MixSIAR relative contributions for node M3.

## Conclusion

In both the longitudinal example based on geochemical tracers and the distributed example based on compound specific stable isotope tracers, D-MixSIAR provided a different posterior distribution of sediment source contributions at the outlet of the defined study watersheds compared to conventional pooled MixSIAR. Field observations and local environmental knowledge suggest that the D-MixSIAR outputs are a more credible assessment of potential sediment sources in these systems supported by lower uncertainties and quicker model convergence.

In the longitudinal study, sediment yield from topsoil erosion was the dominant signal overall in both model outputs but it was only when specific topsoil sources were structured by watershed zone that more constrained source signatures emerged, i.e. organic versus conventional cultivation and permanent versus rotational pasture, permitting refined sub-watershed-specific results. In the distributed example, sediment yield from both forest types was important in pooled and D-MixSIAR and likely to be a function of surface erosion linked to disturbance of the soil during leaf litter collection by local communities and livestock trampling. Additionally, steep topography with dense tributaries and intersecting trackways means slopes are well connected to streams. D-MixSIAR, however, specifically distinguished broadleaf from mixed forests at the outlet because it was informed by the tributary proportions within the deconvolution process. Whereas pooled MixSIAR apparently overestimated broadleaf forest inputs, D-MixSIAR used stratified source samples to weight mixture proportions by sub-watershed; hence, downstream unmixing is constrained by results for upstream sub-watershed inputs, in the context of the key methodological assumptions articulated earlier.

In applications of D-MixSIAR, the source and mixture sampling strategy has to go hand-in-hand with the structural hierarchy of the drainage and sub-watershed network. A key assumption of the approach, in addition to the established need for source sample spatial representativeness, is that the sediment sampled at each node is spatially and temporally representative of the upstream contributing area for the time window of study i.e. the signature of sediment mixtures at a node may change with temporal dynamics of primary source contributions through a hydrological year. Apportionment to sub-watersheds is hence analogous to sediment load with an assumption of mass conservation in terms of source proportions to downstream mixtures. In this regard, user confidence in sample representativeness is paramount and sample numbers and/or mode of sample collection are critical considerations. The benefits of the stratified approach taken by D-MixSIAR in exploring complex systems introduces new sampling and analytical demands that users need to consider against specific research questions. As with other tracer studies, information of sediment load would be an advantage in interpreting the proportional data^61,62^.

We propose that the D-MixSIAR approach offers better system representation than current source apportionment approaches. The demonstration data presented, support distinct advantages of stratification of primary sources by sub-watershed within the D-MixSIAR model framework which (1) reduces the complexity of source groups contributing to high order stream mixture nodes by stratifying primary sources by sub-watershed, (2) decreases variability within sources by removing the duplication effect of tracer values and potential overlapping of source signatures and (3) appropriately weights sub-watershed specific primary source contributions by systematically accounting for sub-watershed contributions. In conventional mixing models at the larger watershed scale (analogous to pooled MixSIAR), having multiple sources with overlapping source tracer composition significantly increases model output uncertainty. The step-change proposed here in stratifying data according to watershed hierarchy, followed by deconvolution at the next level, clarifies this and leads to a model structure that represents better the soil-sediment continuum.

The approach further highlights the need and advantages of proper attention to data hierarchy in river basin systems. This new tool for source apportionment offers wider application across complex environmental systems affected by natural and human-induced change and the lessons learned are relevant to source apportionment applications in other disciplines. The new data handling routines provide evidence to support management of complex human-environment interactions and help tackle the global challenge of improved food, water and energy security.

## Electronic supplementary material

Supplementary Information

## Data Availability

All data generated or analysed during this study are included in this published article and its Supplementary Information files: Datafile text and model code for demonstration examples.
