# Supplementary material for: A deconvolutional Bayesian mixing model approach for river basin sediment source apportionment
Source: Sci Rep. 2018 Aug 30;8:13073. doi: 10.1038/s41598-018-30905-9 (PMC6117284; doi:10.1038/s41598-018-30905-9)
Supplement: Supplementary file 1 — Supplementary Information [file 41598_2018_30905_MOESM1_ESM.pdf]

Supporting Information 1: MixSIAR demonstration data and R code files

Manuscript title: A hierarchical Bayesian mixing model approach for river basin sediment source apportionment

William H. Blake[1*], Pascal Boeckx[2†], Brian Stock[3], Hugh G. Smith[4], Samuel Bodé[2], Hari R. Upadahayay[2], Leticia Gaspar[5], Rupert Goddard[1], Amy T. Lennard[2], Ivan Lizaga[5], David Lobb[6], Philip N. Owens[7], Ellen L. Petticrew[7], Zouzou Kuzyk[6], Bayu D. Gari[8], Linus Munishi[9], Kelvin Mtei[9], Amsalu Nebiyu[8], Lionel Mabit[10], Ana Navas[5] and Brice Semmens[2]

[†]joint first author

Contact authors: william.blake@plymouth.ac.uk; pascal.boeckx@UGent.be

21 pages of SI material – data files and model code.

1. *Filename: source.m1.csv*

Sources,MeandC22,SDdC22,MeandC24,SDdC24,MeandC26,SDdC26,MeandC28,SDdC28,MeandC30,SDdC30,MeandC32,SDdC32,ConcdC22,ConcdC24,ConcdC26,ConcdC28,ConcdC30,ConcdC32,n

BLF,-33.30444323,1.061421351,-32.53722312,1.341995317,-32.02904807,1.205834537,-32.41381423,0.823458785,-33.58704551,0.963452015,-34.3743685,1.275103027,0.180163859,0.330856836,0.250393758,0.245630718,0.225444159,0.112228522,11

LL,-30.0126901,1.098168761,-28.47828514,1.333443057,-27.74104435,1.825569612,-28.04967132,1.666079102,-29.14979688,1.781485188,-28.21568383,1.726309642,0.11262467,0.247950769,0.186150679,0.218650273,0.186121627,0.111283217,11

MF,-29.66694343,2.662938106,-28.32919947,2.681472881,-27.6862613,2.939014362,-29.31221965,1.973734161,-30.92528619,1.498237647,-31.26144861,1.379372987,0.194224188,0.258047837,0.184756889,0.193331316,0.219608311,0.159517727,8

UP,-29.65989513,1.928831334,-28.5751847,2.300541221,-28.042259,2.639705126,-29.13310715,2.265947682,-30.49179496,2.299109239,-30.18853029,2.33566559,0.089421089,0.168845173,0.124306646,0.13015486,0.117125653,0.069976017,12

2. *Filename: source.m2.csv*

Sources,MeandC22,SDdC22,MeandC24,SDdC24,MeandC26,SDdC26,MeandC28,SDdC28,MeandC30,SDdC30,MeandC32,SDdC32,ConcdC22,ConcdC24,ConcdC26,ConcdC28,ConcdC30,ConcdC32,n

LL,-29.83809718,0.442986526,-29.39472818,1.297948238,-28.57990264,2.099674158,-27.87240151,1.54296758,-29.39428161,1.830470909,-28.64702452,2.197381408,0.091386971,0.23690445,0.178185377,0.181810688,0.124610695,0.064541018,2

MF,-29.54796225,2.255413233,-29.55456062,1.747396852,-29.07716603,2.620358836,-29.51616093,2.413119624,-30.90644988,2.303848698,-31.74340961,1.898291917,0.179891828,0.249019014,0.174300346,0.158731889,0.145242858,0.067007881,6

UP,-29.08276946,0.597827209,-28.02751579,0.535549729,-26.80971219,0.639036759,-27.75000318,0.214576998,-29.57445742,0.631988402,-29.23020183,0.941356357,0.069653673,0.145828899,0.095753805,0.115839863,0.116496055,0.080661473,3

3. *Filename: source.m3.csv*

Sources,MeandC22,SDdC22,MeandC24,SDdC24,MeandC26,SDdC26,MeandC28,SDdC28,MeandC30,SDdC30,MeandC32,SDdC32,ConcdC22,ConcdC24,ConcdC26,ConcdC28,ConcdC30,ConcdC32,n

mix.1,-31.08964966,0.101453989,-31.19523537,0.195801009,-31.67603503,0.128689293,-32.31748916,0.077638973,-34.0793241,0.367725172,-

33.80349635,0.2478541,0.288106559,0.4915495,0.344470047,0.326959725,0.301700676,0.160902881,9

mix.2,-31.33428602,0.194960689,-30.86294371,0.021908044,-31.44000065,0.223576092,-31.73226889,0.15694752,-33.61768692,0.171659093,-34.74364354,0.510602209,0.200122411,0.382436393,0.279607426,0.288559713,0.264624476,0.11867108,8

4. Filename: source.pooled.csv

Sources,MeandC22,SDdC22,MeandC24,SDdC24,MeandC26,SDdC26,MeandC28,SDdC28,MeandC30,SDdC30,MeandC32,SDdC32,Meand13C,SDd13C,ConcdC22,ConcdC24,ConcdC26,ConcdC28,ConcdC30,ConcdC32,ConcdC,n

BLF,-33.30444323,1.061421351,-32.53722312,1.341995317,-32.02904807,1.205834537,-32.41381423,0.823458785,-33.58704551,0.963452015,-34.3743685,1.275103027,-27.67072848,2.369828696,0.180163859,0.330856836,0.250393758,0.245630718,0.225444159,0.112228522,10.90634172,11

LL,-29.98582965,1.012734311,-28.61927637,1.319301846,-27.87009947,1.801076627,-28.02239905,1.586193683,-29.18740991,1.712422077,-28.28204393,1.706477776,-22.27554337,2.800921675,0.109357332,0.246251336,0.184925248,0.212982644,0.176658407,0.104092109,3.150268794,13

MF,-29.6159515,2.40386958,-28.85435425,2.332827444,-28.28236333,2.793247478,-29.39962305,2.085254433,-30.91721349,1.802835863,-31.46800332,1.572177823,-25.78540829,2.129474481,0.188081748,0.254178342,0.180275514,0.17850299,0.187737403,0.11987065,7.834050192,14

UP,-28.67677769,3.034279201,-27.55148425,2.981482758,-26.91454579,2.584815628,-28.10876969,2.615458966,-29.49292412,3.024525208,-29.29644286,2.568979945,-21.28618679,2.417930416,0.09339462,0.183855552,0.130148335,0.131375146,0.119141137,0.072777668,2.327224808,15

5. Filename: mix.1.csv

SN,Season,dC22,dC24,dC26,dC28,dC30,dC32

603,EW,-31.04190724,-31.34488815,-31.82401075,-32.15282334,-33.72126203,-33.30546989

611,MW,-31.2060436,-31.01780482,-31.55582718,-32.24644899,-33.83643114,-33.53111569

612,MW,-31.01995269,-31.16259648,-31.66048521,-32.40036725,-34.50238964,-33.86360625

613,MW,-31.04295269,-31.40530482,-31.8117927,-32.30565124,-33.89915151,-34.01576713

616,LW,-31.04190724,-31.05217982,-31.50878516,-32.35652866,-33.76879537,-33.57041344

533,EW,-32.94021974,-32.77920041,-33.50440194,-33.59241703,-36.4320932,-38.19213623

531,LW,-30.71279676,-30.71441982,-31.94107812,-31.85166725,-34.62486372,-35.53576049

532,LW,-30.8840436,-31.22717982,-31.53804806,-32.1494604,-33.70988078,-34.52828126

619,EW,-32.04136178,-32.40947148,-32.00322824,-33.05026705,-34.80922301,-34.89810619

6. Filename mix.2.csv

```
SN,Season,dC22,dC24,dC26,dC28,dC30,dC32
605,EW,-30.54113451,-30.51363815,-31.0061628,-31.4654675,-33.29579316,-
32.85287335
604,MW,-31.55940724,-30.88551315,-31.60583883,-31.68182715,-33.61593051,-
34.18726129
609,MW,-31.22172542,-30.84176315,-31.52842782,-31.9082354,-33.79021748,-
35.1907835
610,MW,-31.22172542,-30.86155482,-31.18573529,-31.60674413,-33.44691278,-
34.85288583
615,LW,-32.84949814,-32.11250145,-31.81069694,-31.87378761,-32.90322199,-
33.69970445
529,EW,-31.7867969,-31.13010398,-31.11048728,-32.12418064,-33.85604887,-
34.0260621
518,MW,-29.19217234,-28.55834318,-29.47020198,-30.54355284,-31.68593411,-
32.2215
531,LW,-31.39422542,-31.21988815,-32.21296366,-32.70341378,-34.14057022,-
34.32815416
```

7. Filename: mix.3.csv

SN,Season,dC22,dC24,dC26,dC28,dC30,dC32

606,EW,-30.83072542,-30.82509648,-31.66131386,-32.03408569,-33.51260754,-33.41579054

607,MW,-31.51296927,-31.23808083,-32.01368708,-32.10197146,-33.44918896,-33.93187995

608,MW,-31.32208905,-31.34801315,-31.97379605,-32.28751532,-33.93187995,-33.37854205

614,LW,-31.39945269,-31.43447148,-31.82205866,-32.32342025,-33.56481671,-33.2045161

517,LW,-31.12835416,-30.61663042,-31.50307649,-32.28313234,-33.6121477,-33.80774191

539,EW,-31.7867969,-31.11048728,-31.13010398,-32.12418064,-33.85604887,-34.0260621

630,EW,-31.75072542,-31.76988815,-32.1344327,-32.57279171,-34.22321263,-34.17079554

530,EW,-31.50435147,-30.83782575,-31.22657145,-32.52042141,-33.55667548,-34.72796357

8. Filename: discr.all.csv

Sources,MeandC22,SDdC22,MeandC24,SDdC24,MeandC26,SDdC26,MeandC28,SDdC28,MeandC30,SDdC30,MeandC32,SDdC32,Meand13C,SDd13C,ConcdC22,ConcdC24,ConcdC26,ConcdC28,ConcdC30,ConcdC32,ConcdC,n

BLF,0,0,0,0,0,0,0,0,0,0,0,0,0,0,0,0,0,0,0,0,0,11

LL,0,0,0,0,0,0,0,0,0,0,0,0,0,0,0,0,0,0,0,0,0,13

MF,0,0,0,0,0,0,0,0,0,0,0,0,0,0,0,0,0,0,0,0,0,14

UP,0,0,0,0,0,0,0,0,0,0,0,0,0,0,0,0,0,0,0,0,0,15

9. Filename discr.m1.csv

Sources,MeandC22,SDdC22,MeandC24,SDdC24,MeandC26,SDdC26,MeandC28,SDdC28,MeandC30,SDdC30,MeandC32,SDdC32,n

BLF,0,0,0,0,0,0,0,0,0,0,0,0,11

LL,0,0,0,0,0,0,0,0,0,0,0,0,11

MF,0,0,0,0,0,0,0,0,0,0,0,0,8

UP,0,0,0,0,0,0,0,0,0,0,0,0,12

---

10. Filename: discr.m2.csv

Sources,MeandC22,SDdC22,MeandC24,SDdC24,MeandC26,SDdC26,MeandC28,SDdC28,MeandC30,SDdC30,MeandC32,SDdC32,n

LL,0,0,0,0,0,0,0,0,0,0,0,0,3

MF,0,0,0,0,0,0,0,0,0,0,0,0,6

UP,0,0,0,0,0,0,0,0,0,0,0,0,5

---

11. Filename: discr.m3.csv

Sources,MeandC22,SDdC22,MeandC24,SDdC24,MeandC26,SDdC26,MeandC28,SDdC28,MeandC30,SDdC30,MeandC32,SDdC32,n

mix.1,0,0,0,0,0,0,0,0,0,0,0,0,9

mix.2,0,0,0,0,0,0,0,0,0,0,0,0,8

---

12. MixSIAR R code

```
# IMIXSED contribution – D-MIXSIAR demonstration code for use with files provided

# Nepal case study (Upper Chitlang)

# Hari Ram Upadhayay + Brian Stock

# June 2017


# --------------------------------------------------------------------

# First-time MixSIAR user will need to install package

# Install instructions at https://github.com/brianstock/MixSIAR


# Recommend to use GitHub version

# library(R2jags) # check JAGS installed properly

# library(RGtk2) # check GTK+ installed properly

# library(devtools)

# install_github("brianstock/MixSIAR", dependencies = TRUE, build_vignettes = TRUE)


# Can use CRAN version instead (will update from Github version after paper is accepted,
merge_nodes fn added)
```

```
# install.packages("MixSIAR")

library(MixSIAR)
setwd('AS REQUIRED')

# ------------------------------------------------------------------
# Part 1: Deconvolutional MixSIAR

# 1a. Load mixture data
mix.1 <- load_mix_data(filename="mix.1.csv",
                iso_names=c("dC22","dC24","dC26","dC28","dC30","dC32"),
                factors="Season",
                fac_random=FALSE,
                fac_nested=FALSE,
                cont_effects=NULL)

mix.2 <- load_mix_data(filename="mix.2.csv",
                iso_names=c("dC22","dC24","dC26","dC28","dC30","dC32"),
                factors="Season",
                fac_random=FALSE,
                fac_nested=FALSE,
                cont_effects=NULL)

mix.3 <- load_mix_data(filename="mix.3.csv",
                iso_names=c("dC22","dC24","dC26","dC28","dC30","dC32"),
                factors="Season",
                fac_random=FALSE,
                fac_nested=FALSE,
                cont_effects=NULL)

# 1b. Load source data
```

```
source.1 <- load_source_data(filename="source.m1.csv",
                        source_factors=NULL,
                        conc_dep=TRUE,
                        data_type="means",
                        mix.1)

source.2<- load_source_data(filename="source.m2.csv",
                        source_factors=NULL,
                        conc_dep=TRUE,
                        data_type="means",
                        mix.2)

source.3 <- load_source_data(filename="source.m3.csv",
                        source_factors=NULL,
                        conc_dep=TRUE,
                        data_type="means",
                        mix.3)

# 1c. Load discrimination
discr.1 <- load_discr_data(filename="discr.m1.csv", mix.1)
discr.2 <- load_discr_data(filename="discr.m2.csv", mix.2)
discr.3 <- load_discr_data(filename="discr.m3.csv", mix.3)

# 1d. Define model structure and write JAGS model file
m1.filename <- "MixSIAR_model_m1.txt"
m1.resid.err <- TRUE
m1.process.err <- FALSE
write_JAGS_model(m1.filename, m1.resid.err, m1.process.err, mix.1, source.1)

m2.filename <- "MixSIAR_model_m2.txt"
m2.resid.err <- TRUE
```

```
m2.process.err <- FALSE

write_JAGS_model(m2.filename, m2.resid.err, m2.process.err, mix.2, source.2)


m3.filename <- "MixSIAR_model_m3.txt"

m3.resid.err <- TRUE

m3.process.err <- FALSE

write_JAGS_model(m3.filename, m3.resid.err, m3.process.err, mix.3, source.3)


# 1e. Run the JAGS model (advice: run="test" first to check files loaded correctly, then use a longer run to reach convergence)

# jags.m1 <- run_model(run="test", mix.1, source.1, discr.1, m1.filename, alpha.prior=1, m1.resid.err, m1.process.err)

# jags.m2 <- run_model(run="test", mix.2, source.2, discr.2, m2.filename, alpha.prior=1, m2.resid.err, m2.process.err)

# jags.m3 <- run_model(run="test", mix.3, source.3, discr.3, m3.filename, alpha.prior=1, m3.resid.err, m3.process.err)


# Runtime start 2:50pm end

jags.m1 <- run_model(run="long", mix.1, source.1, discr.1, m1.filename, alpha.prior=1, m1.resid.err, m1.process.err)

jags.m2 <- run_model(run="long", mix.2, source.2, discr.2, m2.filename, alpha.prior=1, m2.resid.err, m2.process.err)

jags.m3 <- run_model(run="long", mix.3, source.3, discr.3, m3.filename, alpha.prior=1, m3.resid.err, m3.process.err)


# 1f. Process diagnostics, summary stats, and posterior plots

output_JAGS(jags.m1, mix.1, source.1)

output_JAGS(jags.m2, mix.2, source.2)

output_JAGS(jags.m3, mix.3, source.3)


# 1g. Combine MixSIAR objects frome each node/model into lists (must use mix=, source=, and jags=)

model1 <- list(mix=mix.1, source=source.1, jags=jags.m1)

model2 <- list(mix=mix.2, source=source.2, jags=jags.m2)
```

```
model3 <- list(mix=mix.3, source=source.3, jags=jags.m3)

# Combine models into a single list
models <- list(model1, model2, model3)

# Load merging functions: 'merge_nodes', 'plot_nodes', 'summary_nodes'
source("/home/brian/Documents/Isotopes/Hari_soil_Nepal/data_imixsed_mixsiar/merge_nodes.R")

# Use merging functions
merged <- merge_nodes(models)
library(dplyr)
plot_nodes(merged, save_pdf=F, save_png=T)
res.hierarch <- summary_nodes(merged, save_txt=TRUE, filename="summary_hierarchical.csv")

# Save workspace
save.image("hierarchical.RData")

# ------------------------------------------------------------------------------
# Part 2: Pooled MixSIAR (pool all source samples from both subcatchments)
#   mix.3 already loaded

# 2a. Load source data
source.all <- load_source_data(filename="source.pooled.csv",
                    source_factors=NULL,
                    conc_dep=TRUE,
                    data_type="means",
                    mix.3)

# 2b. Load discrimination
discr.all <- load_discr_data(filename="discr.all.csv", mix.3)
```

```
# 2c. Define model structure and write JAGS model file
all.filename <- "MixSIAR_model_m3all.txt"
all.resid.err <- TRUE
all.process.err <- FALSE
write_JAGS_model(all.filename, all.resid.err, all.process.err, mix.3, source.all)

# 2d. Run the JAGS model
jags.m3all <- run_model(run="long", mix.3, source.all, discr.all, all.filename, alpha.prior=1,
all.resid.err, all.process.err)

# 2e. Process diagnostics, summary stats, and posterior plots
output_JAGS(jags.m3all, mix.3, source.all)

# Save workspace
save.image("pooled.RData")

# ----------------------------------------------------------------------
# Part 3: Summarize results and create table
load("pooled.Rdata")

# 3a. Get posterior draws for each season
post.m3all.EW <- as.data.frame(jags.m3all$BUGSoutput$sims.list$p.fac1[,1,])
head(post.m3all.EW)
colnames(post.m3all.EW) <- source.all$source_names

post.m3all.LW <- as.data.frame(jags.m3all$BUGSoutput$sims.list$p.fac1[,2,])
colnames(post.m3all.LW) <- source.all$source_names

post.m3all.MW <- as.data.frame(jags.m3all$BUGSoutput$sims.list$p.fac1[,3,])
colnames(post.m3all.MW) <- source.all$source_names
```

```
####Adding column of different season
post.mix_EW<-cbind(Season="EW",post.mix_dk_EW )
post.mix_LW<-cbind(Season="LW", post.mix_dk_LW)
post.mix_MW<-cbind(Season="MW", post.mix_dk_MW)
dim(post.mix_EW)

DK_season_comp<-rbind( post.mix_EW, post.mix_LW,post.mix_MW)
dim(DK_season_comp)


library(tidyr)
DK.season_contribution <- DK_season_comp %>% gather(Source,value, 2:5)
head(DK.season_contribution)
write.csv(DK.season_contribution, file="DK.seasons_contribution_170612.csv") #### used file for boxplot and further processing
```

---

13. R Code to deconvolute MixSIAR output

```
# Brian Stock
# July 26, 2016
# Merge results from mixing models run above and below river junction

# 1. Run mixing models on each subcatchment.
#    If Model X mixture (mix.X) is used downstream as a source in Model Y, include mix.X as a source in the source.Y file
# 2. Save mix, source, and jags objects for each model as a list, e.g. model1 = list(mix=mix.1,source=source.1,jags=jags.1).
# 3. Downstream mixtures can use upstream mixtures as sources, e.g. source.3 includes mix.1 and mix.2
# 4. Save all models together in one list, like: models = list(model1, model2, model3)
# Either a) no fixed/random effects or b) the same fixed/random effect at all nodes

merge_nodes <- function(models){
```

```
# Number of models
n.models <- length(models)

# # Check each model has mix, source, and jags lists
# for(m in 1:n.models){
#	if(!identical(names(models[[m]]),c("mix","source","jags"))){
#		stop(paste("*** Error: model ",m," does not contain 'mix', 'source' and 'jags' ***",sep=""))}
#	if(!identical(names(models[[m]]$mix),c("data","data_iso","n.iso","n.re","n.ce","FAC","CE","CE_orig","CE_center","CE_scale","cont_effects","MU_names","SIG_names","iso_names","N","n.fe","n.effects","factors","fac_random","fac_nested","fere"))){
#		stop(paste("*** Error: mix object in model ",m," does not contain correct entries ***",sep=""))}
#	if(!identical(names(models[[m]]$source),c("n.sources","source_names","S_MU","S_SIG","S_factor1","S_factor_levels","conc","MU_array","SIG2_array","n_array","SOURCE_array","n.rep","by_factor","data_type","conc_dep"))){
#		stop(paste("*** Error: source object in model ",m," does not contain correct entries ***",sep=""))}
#	if(!identical(class(models[[m]]$jags),"rjags")){
#		stop(paste("*** Error: model ",m," does not contain 'mix', 'source' and 'jags' ***",sep=""))}
# }

# Get ALL source names (incl mix/nodes), and ORIG source names (no mix/nodes)
source.names.all <- NULL
for(m in 1:n.models) source.names.all <- c(source.names.all,models[[m]]$source$source_names)
source.names.all <- unique(source.names.all)
n.sources.all <- length(source.names.all)
remove <- paste0("mix.",1:n.models)
# remove <- paste0("node",1:n.models)
source.names.orig <- setdiff(source.names.all, remove)
n.sources.orig <- length(source.names.orig)
```

```
models[[1]]$source.names <- source.names.orig

# Get indices of source.names.orig for each model
for(m in 1:n.models) models[[m]]$ind <-
match(source.names.orig,models[[m]]$source$source_names)

# Get node matrix (mix as source to other mix)
node.mat <- array(NA,dim=c(n.models,n.models))
for(m in 1:n.models) node.mat[m,] <- remove %in% models[[m]]$source$source_names

# check if we have fixed/random effect or not
effect <- ifelse(models[[1]]$mix$n.effects==1,TRUE,FALSE)

if(!effect){ # if no fixed/random effect
	for(m in 1:n.models){
		# p.merged indexed same as p.global: [draws,source], but ALL sources in
system
		n.draws <- dim(models[[m]]$jags$BUGSoutput$sims.list$p.global)[1]
		models[[m]]$p.merged <- array(0,dim=c(n.draws,n.sources.orig))
		for(src in 1:n.sources.orig){
			for(src.sub in 1:models[[m]]$source$n.sources){
				# do we have a mix node?
				 if(length(grep("mix",
models[[m]]$source$source_names[src.sub]))==1){
					node <- TRUE
					mix.no <- as.numeric(gsub("mix.", "",
models[[m]]$source$source_names[src.sub]))
				 } else { node <- FALSE }
			#	if(length(grep("node",
models[[m]]$source$source_names[src.sub]))==1){
			#		node <- TRUE
			#		mix.no <- as.numeric(gsub("node", "",
models[[m]]$source$source_names[src.sub]))
```

```
#			} else { node <- FALSE }


			if(!node){

				# if(src==models[[m]]$ind[src.sub])
models[[m]]$p.merged[,src] <- models[[m]]$p.merged[,src] +
models[[m]]$jags$BUGSoutput$sims.list$p.global[,models[[m]]$ind[src]]

				if(!is.na(models[[m]]$ind[src]))
{if(src.sub==models[[m]]$ind[src]) models[[m]]$p.merged[,src] <- models[[m]]$p.merged[,src] +
models[[m]]$jags$BUGSoutput$sims.list$p.global[,src.sub]}

			}

			if(node){

				# tmp <-
models[[m]]$jags$BUGSoutput$sims.list$p.global[,src.sub] *
models[[mix.no]]$jags$BUGSoutput$sims.list$p.global[,models[[mix.no]]$ind[src]]

				# models[[m]]$p.merged[,src] <-
models[[m]]$p.merged[,src] + tmp

				tmp <-
models[[m]]$jags$BUGSoutput$sims.list$p.global[,src.sub] * models[[mix.no]]$p.merged[,src]


				models[[m]]$p.merged[,src] <-
models[[m]]$p.merged[,src] + tmp

			}

		}

	}

}

}


if(effect){

	# ASSUMES same fixed effects for ALL nodes, with same levels for all

	# need to write test for this...


	# # test the fixed effect is the same for nodes 1 and 2

	# if(!identical(mix.1$factors,mix.2$factors)){

	#	stop(paste("*** Error: Factors from node 1 and node 2 are not the same
***",sep=""))}
```

```
		# # confirm the indices for each FE/RE level are the same for nodes 1 and 2 (e.g.
season = EW, LW, MW)
		# if(identical(mix.1$FAC[[1]]$labels,mix.2$FAC[[1]]$labels)){
		#	seasons <- mix.1$FAC[[1]]$labels
		#	n.seasons <- length(seasons)
		#	} else {
		#	stop(paste("*** ERROR: fixed/random effect levels not the same for nodes 1
and 2 ***",sep=""))
		# }

		models[[1]]$seasons <- models[[1]]$mix$FAC[[1]]$labels
		n.seasons <- length(models[[1]]$seasons)
		for(m in 1:n.models){
			# p.merged indexed same as p.fac1: [draws,season,source], but ALL sources
in system
			n.draws <- dim(models[[m]]$jags$BUGSoutput$sims.list$p.fac1)[1]
			models[[m]]$p.merged <- array(0,dim=c(n.draws,n.seasons,n.sources.orig))
			for(i in 1:n.seasons){
				for(src in 1:n.sources.orig){
					for(src.sub in 1:models[[m]]$source$n.sources){
						# do we have a mix node?
						 if(length(grep("mix",
models[[m]]$source$source_names[src.sub]))==1){
							node <- TRUE
							mix.no <- as.numeric(gsub("mix.", "",
models[[m]]$source$source_names[src.sub]))
						 } else { node <- FALSE }
						#if(length(grep("node",
models[[m]]$source$source_names[src.sub]))==1){
						#	node <- TRUE
						#	mix.no <- as.numeric(gsub("node", "",
models[[m]]$source$source_names[src.sub]))
```

```
#} else { node <- FALSE }

if(!node){
if(!is.na(models[[m]]$ind[src])) {if(src.sub==models[[m]]$ind[src]) models[[m]]$p.merged[,i,src] <- models[[m]]$p.merged[,i,src] + models[[m]]$jags$BUGSoutput$sims.list$p.fac1[,i,src.sub]}
}
if(node){
# tmp <- models[[m]]$jags$BUGSoutput$sims.list$p.fac1[,i,src.sub] * models[[mix.no]]$jags$BUGSoutput$sims.list$p.fac1[,i,models[[mix.no]]$ind[src]]
tmp <- models[[m]]$jags$BUGSoutput$sims.list$p.fac1[,i,src.sub] * models[[mix.no]]$p.merged[,i,src]

models[[m]]$p.merged[,i,src] <- models[[m]]$p.merged[,i,src] + tmp
}
}
}
} # end season
} # end models
} # end if(effect)

return(models)
}

# 'merged' is result of 'merge_nodes' function
plot_nodes <- function(merged,save_pdf=FALSE,save_png=TRUE){
n.models <- length(merged)
effect <- ifelse(merged[[1]]$mix$n.effects==1,TRUE,FALSE)

if(!effect){ # if no fixed/random effect
n.sources <- dim(merged[[1]]$p.merged)[2]
```

```
		n.draws <- dim(merged[[1]]$p.merged)[1]
		col=RColorBrewer::brewer.pal(n.sources,"Set1")
		for(m in 1:n.models){
			dev.new()
			df <- data.frame(sources=rep(NA,n.draws*n.sources),
x=rep(NA,n.draws*n.sources))  # create empty data frame
			for(src in 1:n.sources){
				df$x[seq(1+n.draws*(src-1),src*n.draws)] <-
as.matrix(merged[[m]]$p.merged[,src]) # fill in the p.global values
				df$sources[seq(1+n.draws*(src-1),src*n.draws)] <-
rep(merged[[1]]$source.names[src],n.draws)  # fill in the source names
			}

			# handle case where a source is not present in this node
			df[df == 0] <- NA
			y <- df %>% group_by(sources) %>% summarize(mean=mean(x)) %>%
mutate(n=1:n.sources) %>% filter(mean>0) %>% select(n)

			my.title <- paste0("Node ",m)
			print(ggplot2::ggplot(df, ggplot2::aes(x=x, fill=sources, colour=sources)) +
				ggplot2::geom_density(alpha=.3, ggplot2::aes(y=..scaled..)) +
				ggplot2::xlim(0,1) +
				ggplot2::theme_bw() +
				ggplot2::xlab("Proportion of Diet") +
				ggplot2::ylab("Scaled Posterior Density") +
				ggplot2::labs(title = my.title) +
				ggplot2::scale_fill_manual(values=col[y$n]) +
				ggplot2::scale_colour_manual(values=col[y$n]) +
				ggplot2::theme(legend.position=c(1,1), legend.justification=c(1,1),
legend.title=ggplot2::element_blank()))

			# Save plot as PDF
			if(save_pdf){
```

```
                    mypath <- paste0("p.node",m,".pdf")  # svalue(plot_post_name),
factor1_names
                    dev.copy2pdf(file=mypath)
                }

                # Save plot as PNG
                if(save_png){
                    mypath <- paste0("p.node",m,".png") # svalue(plot_post_name),
factor1_names
                    dev.copy(png,mypath)
                }
            } # end loop over nodes/models
        } # end p.global posterior plots

        if(effect){ # if we have a fixed/random effect
            n.seasons <- dim(merged[[1]]$p.merged)[2]
            n.sources <- dim(merged[[1]]$p.merged)[3]
            n.draws <- dim(merged[[1]]$p.merged)[1]

            # get colors in advance to keep consistent even when a source is not present for a
given node
            col=RColorBrewer::brewer.pal(n.sources,"Set1")

            for(m in 1:n.models){
                for(f1 in 1:n.seasons){
                  dev.new()
                  df <- data.frame(sources=rep(NA,n.draws*n.sources),
x=rep(NA,n.draws*n.sources))  # create empty data frame
                  for(src in 1:n.sources){
                    df$x[seq(1+n.draws*(src-1),src*n.draws)] <-
as.matrix(merged[[m]]$p.merged[,f1,src]) # fill in the p.fac1[f1] values
                    df$sources[seq(1+n.draws*(src-1),src*n.draws)] <-
rep(merged[[1]]$source.names[src],n.draws)  # fill in the source names
```

```
}

# handle case where a source is not present in this node
df[df == 0] <- NA
y <- df %>% group_by(sources) %>% summarize(mean=mean(x)) %>%
mutate(n=1:n.sources) %>% filter(mean>0) %>% select(n)

my.title <- paste0("Node ",m,": ",merged[[1]]$seasons[f1])  # formerly
factor1_names
print(ggplot2::ggplot(df, ggplot2::aes(x=x, fill=sources, colour=sources)) +
    ggplot2::geom_density(alpha=.3, ggplot2::aes(y=..scaled..)) +
    ggplot2::xlim(0,1) +
    ggplot2::theme_bw() +
    ggplot2::xlab("Proportion of Diet") +
    ggplot2::ylab("Scaled Posterior Density") +
    ggplot2::labs(title = my.title) +
    ggplot2::scale_fill_manual(values=col[y$n]) +
    ggplot2::scale_colour_manual(values=col[y$n]) +
    ggplot2::theme(legend.position=c(1,1), legend.justification=c(1,1),
legend.title=ggplot2::element_blank()))

    # Save plot as PDF
    if(save_pdf){
        mypath <-
paste0("p.node",m,".",merged[[1]]$seasons[f1],".pdf")  # svalue(plot_post_name), factor1_names
        dev.copy2pdf(file=mypath)
    }

    # Save plot as PNG
    if(save_png){
        mypath <-
paste0("p.node",m,".",merged[[1]]$seasons[f1],".png")  # svalue(plot_post_name), factor1_names
        dev.copy(png,mypath)
```

```
                                }
                        } # end p.fac1 posterior plots
                } # end loop over models
        }
}

# 'merged' is result of 'merge_nodes' function
summary_nodes <- function(merged,save_txt=TRUE,filename="summary_nodes.txt"){
        n.models <- length(merged)
        res <- vector("list",n.models)
        effect <- ifelse(merged[[1]]$mix$n.effects==1,TRUE,FALSE)

# open file
cat("
Summary Statistics
",sep="", file=filename, append=FALSE)

        if(!effect){
                for(m in 1:n.models){
                        # calculate stats
                        res[[m]]$quantiles <- round(apply(merged[[m]]$p.merged,2,function(x)
quantile(x,c(.025,.05,.95,.975))),3)
                        res[[m]]$medians <- round(apply(merged[[m]]$p.merged,2,median),3)
                        res[[m]]$means <- round(apply(merged[[m]]$p.merged,2,mean),3)
                        res[[m]]$sds <- round(apply(merged[[m]]$p.merged,2,sd),3)

                        # combine stats
                        res[[m]]$stats <-
rbind(res[[m]]$quantiles,res[[m]]$medians,res[[m]]$means,res[[m]]$sd)
                        rownames(res[[m]]$stats) <-
c(0.025,0.05,0.95,0.975,"median","mean","SD")
                        colnames(res[[m]]$stats) <- merged[[1]]$source.names
```

```
                    out <- capture.output(res[[m]]$stats)

            # print to screen
cat("
# ----------------------------------------------------------------
# Node ",m,"
# ----------------------------------------------------------------
",sep="")
cat(out,sep="\n")

            # print to file
cat("
# ----------------------------------------------------------------
# Node ",m,"
# ----------------------------------------------------------------
",sep="",file=filename, append=TRUE)
cat(out,sep="\n",file=filename, append=TRUE)
            } # end model
      } # end if no effect

      if(effect){
            n.seasons <- dim(merged[[1]]$p.merged)[2]
            for(m in 1:n.models){
                  for(i in 1:n.seasons){
                        # calculate stats
                        res[[m]]$quantiles[[merged[[1]]$seasons[i]]] <-
round(apply(merged[[m]]$p.merged[,i,],2,function(x) quantile(x,c(.025,.05,.95,.975))),3)
                        res[[m]]$medians[[merged[[1]]$seasons[i]]] <-
round(apply(merged[[m]]$p.merged[,i,],2,median),3)
                        res[[m]]$means[[merged[[1]]$seasons[i]]] <-
round(apply(merged[[m]]$p.merged[,i,],2,mean),3)
```

```
                                        res[[m]]$sds[[merged[[1]]$seasons[i]]] <-
round(apply(merged[[m]]$p.merged[,i,],2,sd),3)

                                        # combine stats
                                        res[[m]]$stats[[merged[[1]]$seasons[i]]] <-
rbind(res[[m]]$quantiles[[i]],res[[m]]$medians[[i]],res[[m]]$means[[i]],res[[m]]$sd[[i]])
                                        rownames(res[[m]]$stats[[i]]) <-
c(0.025,0.05,0.95,0.975,"median","mean","SD")
                                        colnames(res[[m]]$stats[[i]]) <- merged[[1]]$source.names
                                        out <- capture.output(res[[m]]$stats[[i]])

                    # print to screen
cat("
# ---------------------------------------------------------------
# Node ",m,": ",merged[[1]]$seasons[i],"
# ---------------------------------------------------------------
",sep="")
cat(out,sep="\n")

                    # print to file
cat("
# ---------------------------------------------------------------
# Node ",m,": ",merged[[1]]$seasons[i],"
# ---------------------------------------------------------------
",sep="",file=filename, append=TRUE)
cat(out,sep="\n",file=filename, append=TRUE)
                              } # end season
                    } # end model
          } # end if(effect)
          return(res)
}
```

Supporting Information 2: Full Geochemical data

as .csv file

GROUP,Na,Mg,Al,Si,P,S,Cl,K,Ca,Ti,Cr,Mn,Fe,Co,Ni,Cu,Zn,Ga,Br,Rb,Sr,Zr,Nb,Ba,Ce,Pb

CB_U,7271,11315,193683,486337,2944,7569,167,36112,6717,6730,157,387,64972,108,104,46,166,
30,28,194,123,267,23,630,421,99

CB_U,7250,12462,207653,491024,3127,3373,230,37424,6322,6681,147,1112,86560,124,95,75,207,
31,38,208,128,271,23,269,362,115

CB_U,7281,12169,204132,484160,3122,3140,177,36963,6324,6676,148,1058,86556,121,97,56,198,
29,42,216,125,278,26,280,305,97

CB_U,6667,9942,212549,506157,2691,1229,96,37240,3110,6762,136,2023,90116,113,81,56,131,31,
48,218,117,287,30,590,373,45

CB_U,7355,12118,197367,497769,4288,2942,203,35802,6996,6843,144,792,75113,25,98,64,164,37,
32,203,122,288,29,911,348,90

CB_U,7062,10069,215078,510680,2694,1123,123,37577,3216,6935,144,2098,92237,126,82,43,131,
27,43,212,132,301,26,316,351,91

CB_U,7304,11527,204415,511931,2714,2410,273,37143,1693,6832,146,729,77657,106,111,52,147,
23,65,206,113,279,27,227,324,121

CB_U,7538,9701,198283,487333,3208,3497,249,37274,5021,6528,143,714,77558,86,117,48,185,31,
42,205,124,281,24,247,362,101

CB_U,6810,10218,204143,489159,3865,2530,185,36030,1946,6741,157,1869,87491,138,129,61,149
,34,65,201,125,280,28,438,280,74

CB_U,6267,9009,209108,481742,3588,2332,199,38360,1300,6458,120,2098,89244,25,89,53,126,25,
85,225,130,273,24,534,324,92

CB_U,5651,9117,204583,471919,3618,2483,239,37609,4463,6442,131,2642,93756,144,91,75,164,2
5,83,224,125,265,22,580,352,151

CU_U,9614,13980,205199,454997,8801,2683,131,28843,6870,10869,142,3298,105486,139,89,90,20
2,34,43,170,134,307,38,220,272,125

CU_U,10423,14904,193933,454610,8251,2702,144,26407,6644,11253,109,3519,96657,172,102,92,2
16,17,35,158,139,319,36,232,311,74

CU_U,8908,14060,205458,459950,8010,2628,105,29558,6667,10590,137,3528,100322,147,96,100,1
85,31,50,183,124,296,28,445,294,81

CU_U,6586,10235,211360,482014,6126,2397,106,34484,6768,8408,127,3075,90470,131,91,73,185,
37,44,216,132,288,29,738,406,61

CU_U,6694,10133,219740,471839,6756,2418,106,36226,6443,8813,142,3117,95017,140,64,85,173,
38,43,210,133,269,30,585,360,77

CU_U,5810,9390,221156,485615,5425,2123,93,38024,5696,7532,153,3175,91562,100,81,97,169,34,
34,233,140,261,28,571,333,80

CU_U,9699,12634,213182,453684,7628,2395,116,31253,6970,11527,137,3365,104713,164,101,108,230,33,34,172,134,298,36,702,284,169

CU_U,6794,10335,221278,461164,7134,2531,128,37819,6702,9163,132,3339,98520,141,80,47,209,38,44,224,129,256,32,214,312,90

CU_U,6265,8999,217054,484316,5558,2142,113,37800,5943,7865,105,3045,92406,123,88,84,164,27,46,235,134,274,26,698,365,110

CU_U,11657,12394,183272,465200,7870,2143,109,20431,11894,14376,135,1741,129107,189,91,74,157,28,41,87,216,398,35,213,305,63

CU_U,10801,15878,184524,462524,6863,2100,142,18725,7571,14668,182,1831,132481,188,153,47,168,30,44,100,194,387,37,240,246,69

CU_U,11143,19919,186506,446042,7663,2209,140,16971,9989,13985,110,2209,134965,25,81,80,162,23,46,86,230,353,31,221,278,56

CU_U,10233,13297,184538,451892,10243,2321,114,20008,9163,15203,108,2562,138163,224,112,70,166,35,50,105,179,412,39,220,245,70

CU_U,9443,16929,186930,461752,7251,2007,106,18017,6607,15531,114,2231,137355,207,117,73,182,33,48,88,153,410,41,188,335,48

CU_U,8572,17183,192066,444162,8057,2206,135,18884,8429,14756,152,2419,140485,214,110,87,189,28,49,100,197,366,35,196,255,57

PP_U,5881,10677,206206,495194,5693,2324,145,36732,5510,6899,159,2475,89291,25,114,56,163,27,35,206,115,293,22,467,364,77

PP_U,5583,4969,196487,496067,3987,3189,199,33122,5971,6488,157,1206,66839,100,67,91,147,28,37,219,219,260,22,208,316,98

PP_U,5570,4979,200230,501829,3953,3140,200,34123,5599,6690,157,1051,60470,108,59,73,112,29,34,224,216,262,19,221,429,104

PP_U,5829,5809,215379,499261,3498,2658,136,37234,5324,7067,184,1295,67474,118,78,57,114,38,37,234,215,250,27,252,303,91

PP_U,6260,6853,213354,524972,5241,2236,161,39391,5626,5585,167,951,72562,128,79,74,214,27,20,216,191,212,20,593,319,91

PP_U,7908,11820,217975,505055,4949,2548,169,36311,4260,6791,157,1194,85238,140,119,59,182,29,24,210,166,249,26,267,306,80

PP_U,7681,11295,204822,497988,6537,3047,196,33555,5319,6606,149,1325,84992,120,120,70,197,31,27,191,162,252,23,249,393,74

RM_U,8113,19170,175372,478714,4487,5967,324,32568,23665,5786,171,1348,85998,114,116,58,172,21,15,172,168,294,29,229,284,93

RM_U,10245,18179,149225,479770,3848,7930,325,26972,31655,6724,124,1016,87306,94,93,58,219,26,6,146,167,359,35,218,222,82

RM_U,10154,20246,147667,450928,4650,12151,261,29658,40676,6646,151,1266,87907,96,100,51,216,25,8,154,162,433,43,814,338,89

RM_U,10860,19085,147500,457622,4546,10942,219,30955,33817,6565,138,1104,83559,25,116,74,221,25,9,154,166,442,48,577,257,76

RM_U,13285,19648,158898,498462,4778,4624,175,19928,16069,17692,162,1391,128788,195,93,52,152,18,6,93,219,477,53,549,328,53

RM_U,16831,34849,182163,463001,3435,2246,110,19797,10872,18281,178,2039,163700,253,157,61,157,21,10,74,222,357,38,234,267,44

RM_U,10128,20050,150419,449975,4789,11521,317,30451,40732,6332,119,1304,89318,119,105,70,229,33,17,165,166,395,41,870,299,93

RM_U,16338,30199,169556,472238,3594,2082,109,18365,10311,21860,221,1876,159887,270,115,57,171,25,4,74,219,449,47,194,290,32

RM_U,15535,18317,146237,516604,4571,3316,162,16626,11974,19591,181,1410,126017,192,108,45,165,22,7,69,220,552,45,213,295,42

RM_U,15535,18317,146237,516604,4571,3316,162,16626,11974,19591,181,1410,126017,192,108,45,165,22,7,69,220,552,45,213,295,42

RP_U,7443,14390,202605,463703,6556,2735,137,29233,4923,10951,136,2778,103607,145,96,61,213,30,56,173,112,325,31,460,295,77

RP_U,7312,14489,201901,460513,6490,2698,118,29117,4948,10726,147,2730,101975,146,114,70,204,29,53,174,120,312,34,195,275,100

RP_U,8546,16924,201806,455572,7387,2856,133,26589,4943,11888,140,2687,103733,162,111,58,213,34,53,163,133,331,38,232,272,75

RP_U,9960,15539,196998,438973,8100,2939,151,23112,5322,12822,133,2870,110623,137,97,96,198,37,58,141,125,345,41,231,257,118

RP_U,10226,19537,185859,447480,9139,3086,118,20922,6607,13415,127,2777,109707,175,97,86,238,23,53,132,116,379,44,437,210,103

RP_U,9003,16344,180391,463371,8094,3241,170,23812,5938,11538,130,2302,97119,146,53,48,172,21,59,142,106,382,39,194,284,108

RP_U,6926,15374,188951,495809,5787,2704,115,29039,5106,9529,122,1401,82151,110,84,71,162,23,42,163,102,363,31,231,307,58

RP_U,9061,15014,188683,451215,8551,3048,172,26454,8459,11067,126,3134,107166,129,93,74,217,32,50,155,118,320,31,794,279,94

RP_U,10403,14235,200310,448137,7387,2681,127,26269,5528,12093,119,3564,113756,173,94,59,192,31,49,153,123,333,39,226,237,147

RP_U,9516,12555,173198,487484,7530,2781,306,29072,8953,10851,107,3236,85100,140,75,71,161,32,34,151,103,369,35,203,215,104

MIX1,6415.4,12422.9,193101.3,466925.8,6605.4,4725.7,177.4,34895.7,21094.8,6834.2,174.2,2069.9,85962.9,144.4,128.6,108,365.4,20,69.4,190.5,172.3,269.9,29.4,252.7,398.1,154.1

MIX1,6419.8,12371.9,193647.3,464069.2,6568.6,4874.3,174.6,35064.2,21255.1,6755.7,165.7,2106.7,86075.1,128.3,87.4,102.2,343.3,34.5,63.9,190.5,175.1,273.3,27,252.8,302,143.3

MIX1,5863.3,10563.2,186925.1,449282.7,5946.5,4526.5,165.5,35939.6,18950.8,6431.6,173.6,2845.3,86209.1,134.4,133,90.9,252.6,33,82.4,215.3,196.4,244.6,29.3,737.4,346.6,117.4

MIX1,7408.4,12627.6,199276.7,471227.2,6460.5,4579,151.3,34972.5,19551.1,6992.3,135,2130.8,88837.2,141.4,116.4,77.2,264.9,28,34.2,194.2,172.6,280.4,26.4,764.1,268.3,127.7

MIX1,6925.7,12724,197405,470096.8,6259,4501.1,159.3,34893.6,19944,7003.7,148.8,2193.2,88440.8,91,101.9,80.1,271.1,30.6,33.8,182.1,171.9,271.5,25.4,821.9,322.2,119.8

MIX1,6656.4,13193.8,196106.9,470646.5,6528.8,4462.8,193,35725.5,13745.1,7019.8,145.9,2411.3,86717.9,135.8,100.4,84,269,27.8,56.8,193,159.6,260,28.6,609.8,336.6,99.8

CB_L,7313,9939,207550,517602,2556,1784,170,35538,5847,6634,138,940,82341,109,91,48,175,32,34,193,178,279,23,201,322,105

CB_L,7655,11728,195439,499005,3535,3482,263,33717,12930,6542,165,1194,83895,25,123,69,188,35,40,187,185,290,23,229,386,109

CB_L,6846,10552,195085,494227,3975,3039,142,34001,11761,6579,173,1837,88961,133,121,85,201,26,36,177,199,262,26,220,55,75

CB_L,6857,6509,214118,526865,2223,898,124,36710,3291,5909,169,617,89509,129,122,48,135,29,15,210,211,273,20,416,313,68

CB_L,6977,9974,202670,499581,3248,2415,195,35733,8272,6659,155,1234,81522,118,111,62,172,27,36,192,173,277,21,502,330,114

CB_L,6803,14067,196923,487922,3565,2674,123,33697,14141,5904,191,1366,87369,114,164,70,185,25,44,200,195,232,20,226,260,153

CB_L,7118,11663,206208,506496,2712,1532,128,32833,3131,6008,177,1452,93412,129,168,62,168,25,42,192,185,226,20,668,224,94

CB_L,6539,7673,215999,498792,2769,1597,119,35680,2430,5766,199,2019,94517,172,139,58,182,30,27,210,191,251,19,219,330,94

CB_L,7898,9325,228605,567105,1337,1945,119,38634,4203,6190,163,148,35708,58,94,52,136,26,8,210,214,263,22,594,313,59

CB_L,7760,9164,225911,562047,1378,1967,94,38111,4341,6226,173,125,35606,60,112,53,128,33,4,212,209,274,18,868,321,61

CB_L,6825,10281,196808,495818,4482,3094,159,34876,11271,6548,145,1746,87792,142,94,68,222,24,44,194,191,270,21,529,321,111

CB_L,6882,6084,209718,519694,2029,999,118,36193,3904,5847,165,1227,97059,154,90,42,153,24,17,204,205,253,24,681,318,100

CU_L,6606,12607,213167,517384,3299,1911,105,35180,5798,6088,139,665,78504,125,107,81,155,33,18,208,183,243,22,503,294,93

CU_L,7600,11301,210735,522848,2042,3587,121,34664,7580,6434,184,301,69700,92,120,0,157,29,16,193,185,268,22,244,306,83

CU_L,6416,14171,206662,500888,3748,2019,90,36497,6059,6051,139,753,78987,25,102,90,188,27,27,221,170,244,23,258,273,109

CU_L,6161,14155,204987,492057,5075,2852,131,36416,10589,6005,199,1070,80946,123,126,101,204,29,44,227,181,245,24,234,284,92

CU_L,6919,11172,218555,488401,6778,2375,88,38545,9435,6483,183,1673,89942,117,108,77,192,37,31,241,203,244,26,425,392,75

CU_L,6566,11019,214120,487871,6585,2496,114,37541,9581,6326,180,1744,88872,120,107,92,162,32,31,230,194,252,20,561,328,81

CU_L,6710,10749,211433,490267,7142,2856,115,36802,10300,6239,222,1701,87026,93,103,63,171,33,33,224,206,248,19,442,361,82

CU_L,6630,11417,217311,479842,7643,2818,119,38684,9227,6474,154,1727,89803,25,113,69,205,26,30,231,208,246,23,492,293,90

CU_L,6617,11020,212923,487730,7519,2694,135,37757,10124,6464,164,1777,88683,119,101,64,190,36,38,228,196,246,23,281,306,126

CU_L,6693,11099,220025,483405,6672,2603,121,38895,7395,6485,180,1527,91914,130,121,77,186,39,36,240,210,242,20,288,261,131

CU_L,6590,9457,215758,491679,6421,2500,135,38418,8204,6358,175,1613,88312,138,111,65,182,27,37,245,208,241,25,500,348,124

CU_L,6540,10856,214619,484047,8013,2732,100,37684,9295,6403,184,1692,89238,127,116,72,167,35,30,231,209,242,28,246,296,80

CU_L,6624,12350,218122,486474,6749,2296,104,38891,8165,6594,185,1734,91844,158,106,54,169,33,31,240,205,257,28,511,346,143

CU_L,6728,13765,218038,484532,5842,2102,153,39934,6759,6258,166,1741,86822,99,108,86,196,28,32,250,179,237,19,637,315,87

CU_L,6387,10925,214450,496391,5599,1951,111,38606,6424,6476,143,1763,81944,25,92,92,183,35,34,235,180,252,23,672,365,97

CU_L,6581,9021,213120,513458,5208,1936,112,37684,5323,6407,155,1423,76212,101,97,56,154,36,29,223,194,265,22,266,430,86

CU_L,6674,12224,217799,491566,4949,1951,142,39584,9289,6376,167,1627,84579,25,90,70,162,24,37,236,172,242,24,242,402,92

CU_L,6220,11855,217906,477876,6004,2150,110,38556,6290,6397,184,1679,89485,134,92,86,181,38,37,246,177,243,22,254,282,138

CU_L,6432,12398,220367,470985,6223,2155,152,39371,5645,6457,184,1658,91866,130,137,78,177,31,36,239,180,237,24,260,422,89

RP_L,4620,11992,153208,517933,8206,4197,188,34423,10231,5152,110,1716,57417,68,89,95,208,24,28,171,125,351,20,473,332,83

RP_L,4358,11978,170795,514967,6382,3166,141,33945,6267,5963,204,2351,67367,87,126,94,202,21,30,166,111,331,19,205,110,105

RP_L,5147,11740,164500,518031,6561,3321,143,29995,6720,5581,147,2337,67203,93,103,78,211,20,26,166,119,375,26,201,270,56

RP_L,6299,20788,170511,550314,4229,1450,103,32370,4423,6106,133,1025,76029,25,126,71,140,23,26,177,57,312,28,227,255,62

RP_L,7151,20788,166556,549135,4582,1506,112,31323,4991,6000,157,1015,72683,25,106,77,147,28,26,169,61,353,25,508,262,83

RP_L,6194,16857,164742,572512,3690,1278,109,31274,4575,5767,150,995,66038,25,93,64,144,21,27,165,75,396,23,513,354,78

RP_L,5138,11328,156783,507746,5427,3838,173,28504,7319,5598,123,3134,70368,25,117,55,179,26,36,155,103,384,21,585,281,81

RP_L,5207,10719,153010,522412,4628,3402,158,28195,5713,5538,155,2231,65770,115,109,54,175,18,44,150,96,386,21,213,296,93

RP_L,6736,9331,199704,472417,6993,3976,154,34738,5162,6673,193,1411,74428,112,123,48,190,34,31,210,166,257,22,600,308,101

RP_L,6845,9981,203310,466612,6131,3285,146,35576,4258,6758,201,1542,84775,25,109,70,198,36,40,221,169,239,24,618,55,125

RP_L,6742,9475,203650,471381,5095,3299,151,34654,3840,6523,210,1398,82078,25,107,68,194,33,54,208,168,253,22,479,292,86

RP_L,7176,9764,198016,479609,6661,3818,132,35686,7959,6660,164,1306,71366,93,101,70,177,32,23,218,205,264,24,512,339,87

RP_L,7089,9565,196641,485030,6130,4134,156,35134,5893,6532,164,959,67157,80,111,71,155,43,30,200,187,265,20,240,321,63

RP_L,7248,9692,200129,472298,6517,4397,174,36351,6739,6884,173,1145,71299,119,108,66,189,38,36,214,212,252,25,216,293,110

RP_L,6273,9093,209624,504480,4883,2091,96,34925,4972,6749,142,1857,83630,25,75,74,145,28,39,216,124,289,24,622,311,54

RP_L,6465,9578,198947,506789,4870,2350,146,34038,4934,6917,148,2003,83436,134,80,47,166,28,40,193,121,300,27,449,271,69

RP_L,6263,9654,187731,470756,8936,3885,250,35381,5615,6391,157,1455,73750,100,108,85,226,31,26,212,224,298,21,245,356,80

RP_L,6332,9828,199630,478763,7945,3198,185,36357,4139,6567,155,1302,75455,120,93,73,195,33,26,223,229,286,22,425,319,102

RP_L,6738,7572,191680,512545,4524,2901,111,32563,3779,6387,162,713,70354,75,97,56,148,28,29,194,223,293,24,227,256,84

RP_L,6585,7595,190967,510721,4496,2795,108,32748,3874,6413,151,712,69984,104,93,57,141,22,26,196,222,284,22,185,266,63

RM_L,7114,24818,135078,428728,5534,5644,370,25995,81734,5439,171,1722,76010,91,128,138,363,19,16,153,173,335,24,789,163,131

RM_L,6774,16308,136415,456340,3334,7897,305,26064,77134,6409,190,1329,74058,106,95,246,787,21,8,146,175,433,27,268,234,129

RM_L,5670,17178,88967,339786,3138,7360,281,18530,223687,5195,174,1118,51549,25,73,96,274,12,12,121,172,441,21,163,55,81

RM_L,8789,18482,153357,457235,4617,6094,289,27249,53399,6630,139,1190,84428,130,94,66,203,30,10,155,199,318,29,582,224,73

RM_L,9492,16760,163096,470562,5719,6946,219,27334,34363,5917,146,1292,89751,138,106,86,207,27,10,162,185,297,27,435,222,64

RM_L,12611,28729,128592,417850,5262,8687,346,18206,50119,6871,242,1613,92035,136,137,127,655,22,13,106,221,294,24,664,167,168

RM_L,11197,30017,150181,444532,3320,5429,244,23776,74367,7765,124,1571,104830,177,93,90,234,28,4,137,212,295,27,192,195,81

RM_L,11115,19608,170583,505179,3196,5722,169,29863,34676,6621,128,1020,93011,123,124,47,205,22,4,169,163,325,34,602,317,77

RM_L,11551,19708,134604,396083,4400,16785,251,35149,67378,5442,85,1739,89746,137,80,71,266,28,15,228,175,495,74,530,408,97

RM_L,6781,15457,111415,312398,7416,14486,507,27103,112403,5200,158,2195,81042,87,142,118,355,26,47,202,234,397,44,656,279,140

RM_L,7180,20164,127193,438465,3720,8474,279,24542,83768,6269,142,1150,70686,25,73,203,612,17,14,138,170,480,29,531,55,124

RM_L,7144,23942,177768,440062,4558,4706,221,30234,30684,6515,201,1497,88520,127,174,80,185,33,39,179,191,242,19,451,273,130

RM_L,14835,48364,128248,399079,3840,8203,285,13998,72010,7176,266,1992,102312,178,191,115,347,23,8,92,249,277,21,150,201,125

RM_L,8015,51183,79627,237094,2869,5414,303,10285,288635,4767,145,3072,66496,100,108,95,244,20,15,75,243,188,18,111,55,89

RM_L,3625,10523,85648,244253,10069,13151,1219,23523,63342,5862,126,1703,76134,140,147,150,442,31,47,159,242,333,25,121,196,166

RM_L,3560,18259,45132,144163,2248,5378,452,8559,487110,2953,68,2425,37341,50,64,73,159,6,13,54,376,195,9,99,55,55

RM_L,7945,18565,178526,481949,4024,3566,156,33790,29664,5561,134,1085,79047,106,115,45,161,27,11,175,162,276,21,248,287,73

RM_L,8055,19443,166609,447019,4402,5972,311,32225,47223,5674,165,1633,82973,122,119,106,201,26,12,193,178,297,28,551,322,108

RM_L,7764,17562,167659,453591,5231,7021,252,31981,19812,5990,173,1251,85553,118,111,61,190,22,23,189,169,326,31,464,235,107

RM_L,7234,18406,149923,414608,5576,11343,313,29933,23331,5908,162,1152,81577,141,137,91,220,31,29,174,175,314,28,252,257,98

RM_L,6707,18185,158929,406360,5833,13439,228,32379,20666,5617,196,1304,87124,121,144,86,264,30,32,197,179,263,31,209,294,104

MIX2,6572.8,10653.5,203710,483638.9,5168.3,3694.5,181.3,35878,12794.8,6643.8,155.3,2017.1,88660.2,91,124.9,62.2,211.7,30,59.4,196.1,209.8,250.5,22.3,471.8,276.3,82.9

MIX2,6362.3,10368.8,202943.4,482662.6,5263.6,3773.6,211.3,35830.7,12719.3,6591,153.8,1903.7,88058.5,143.4,107.5,78,243.8,29.5,64.2,190.4,218.3,253.5,23.5,286.3,361.1,111.4

MIX2,5933.2,8941.4,196280.9,457844.8,5013,3606.3,154.3,37297,13060.7,6512.9,143.9,2917.4,91651.7,147.6,110.9,88.7,216.7,40.4,89.3,216.8,242,226.5,22.9,499,355,105.2

MIX2,5771.8,9379.7,194329.8,457416.1,5557.6,3882.2,177.7,37069.9,15153.1,6387.1,154.1,3590.6,91661,123.4,109.4,85.3,272.7,33.1,120.5,226.3,219.2,215.8,21.9,659.3,286.6,80

MIX2,6727.1,10965.6,203293.4,484342.4,5861.1,4007.4,176.5,35621.6,12468.1,6595.2,154.6,2241.6,84560,122.1,120,93.9,252.2,29.3,62.2,197,189.2,243.9,23.8,285.6,308.1,111.5

MIX2,6525,11083.5,202965.9,483028.4,5857.2,4086.6,200.4,35742.3,12500.8,6671.4,119.4,2135,85381.1,115.3,93.9,77,261.6,36.6,56.1,190.7,186,237.2,24.4,633.4,339.8,106.2
